# Utilization of long-read sequencing for the detection of structural rearrangements with AgileStructure

**DOI:** 10.1093/bioinformatics/btag294

**Published:** 2026-05-08

**Authors:** Carolina Lascelles, Morag Raynor, Laura A Crinnion, Ailsa M S Rose, Christine P Diggle, James A Poulter, Christopher M Watson, Ian M Carr

**Affiliations:** School of Medicine, University of Leeds, St. James’s University Hospital, Leeds, United Kingdom; School of Medicine, University of Leeds, St. James’s University Hospital, Leeds, United Kingdom; School of Medicine, University of Leeds, St. James’s University Hospital, Leeds, United Kingdom; North East and Yorkshire Genomic Laboratory Hub, St James’s University Hospital, Leeds, United Kingdom; School of Medicine, University of Leeds, St. James’s University Hospital, Leeds, United Kingdom; School of Medicine, University of Leeds, St. James’s University Hospital, Leeds, United Kingdom; School of Medicine, University of Leeds, St. James’s University Hospital, Leeds, United Kingdom; School of Medicine, University of Leeds, St. James’s University Hospital, Leeds, United Kingdom; North East and Yorkshire Genomic Laboratory Hub, St James’s University Hospital, Leeds, United Kingdom; School of Medicine, University of Leeds, St. James’s University Hospital, Leeds, United Kingdom

## Abstract

**Motivation:**

Changes in genome organisation contribute to genetic disease when they disrupt gene function or regulation. Structural rearrangements may interrupt coding sequence or alter expression through promoter loss or gain, chromatin changes, copy-number variation, or disruption of short-range regulatory elements. Although short-read sequencing excels at detecting small variants, it performs poorly at resolving breakpoints of large rearrangements, especially in repetitive or low-complexity regions. Long-read sequencing overcomes these limitations, but analytical tools have not kept pace, making accurate identification and annotation of large structural variants challenging.

**Results:**

We developed AgileStructure, a desktop application for locating and annotating large‑scale genomic rearrangements using aligned long‑read data. The software enables user‑guided exploration of breakpoint‑spanning reads, supporting accurate interpretation of complex events and filling a key gap in current structural variant analysis workflows.

**Availability and implementation:**

Source code, binaries, user guide, and example aligned read data, are available on GitHub: https://github.com/msjimc/AgileStructure. An archived version is also available on Zenodo at https://doi.org/10.5281/zenodo.18610110.

## 1 Introduction

Large structural genomic rearrangements occur due to a range of mechanisms, such as an unsuccessful attempt to repair a chromosomal break, template switching during DNA replication, or nonhomologous recombination between highly homologous but distinct genomic regions (Weckselblatt and Rudd 2015, Burssed *et al.* 2022). These rearrangements may result in the deletion, inversion, translocation or duplication of extended regions of the genome. A 2022 study (Collins *et al.* 2020), analyzed short read sequencing data for 14 891 genomes, found 433 371 structural variants (> 1 kb in length) that were thought to cause approximately 25–29% of all protein truncations and that 3.9% of participants had a variant longer than 1 megabase (Mb). While an analysis of the short-read sequencing data of the 1000 Genomes Project (1000 Genomes Project Consortium *et al.* 2015) revealed the subjects had a median value of 18.4 Mb of DNA affected by a structural rearrangement compared to 3.6 Mb affected by single base changes (Sudmant *et al.* 2015). As these alterations may cause disease due to the disruption of the normal function of genes within or near the rearrangement, they are a frequent cause of morbidity and mortality (Burssed *et al.* 2022).

While the effects of many rearrangements may manifest in the individual in which they arose, others may only impact the patient’s descendants. For example, a balanced translocation may move extended regions of DNA between chromosomes while maintaining gene copy number, structural integrity, and chromatin environment, resulting in normal gene expression and phenotype. However, if they have a child who inherits only one of the derivative chromosomes, they may present with a deleterious phenotype due to the abnormal copy number of genes in the now unbalanced translocation (Davis *et al.* 1985).

Given the importance of rearrangements as drivers of disease, there has been significant interest in their detection and annotation. Assessment of a patient’s karyotype still plays an important diagnostic role in the detection of whole chromosome aneuploidies and interstitial deletions and duplications, such as those that cause Down syndrome, Smith-Magenis syndrome or DiGeorge syndrome (Jenkins *et al.* 2022). Since these tend to cause disease due to the abnormal copy number of many genes, the fact that they are poorly characterized is not necessarily an issue; however, knowledge of the affected genes may inform a patient’s treatment and disease course.

For the detection of smaller rearrangements, microarray-based techniques such as array comparative genomic hybridization (array-CGH) are often used (Grunert *et al.* 2024). These can then be further refined using multiplex ligation-dependent probe amplification (MLPA) to determine the genomic copy number across the rearrangement (Stuppia *et al.* 2012). A major drawback of array-CGH and MLPA is their inability to detect changes that don’t affect copy number and so are blind to balanced translocations and inversions. Similarly, they are unable to define the integration site of transposed sequences and so are of limited use in situations where a gene at or near the site of insertion is disrupted rather than genes present on the duplicated DNA.

More recently, next-generation sequencing (NGS) has revolutionized the detection of single base substitutions and short (approximately less than 150 bp) insertions and deletions. The resultant variant datasets are filtered to identify likely disease-causing variants based on their location, annotation in previous studies, or possible pathogenicity using in silico prediction tools such as PolyPhen (Adzhubei *et al.* 2010) or SIFT (Sim *et al.* 2012). However, despite the development of a number of applications for the detection of structural rearrangements, such as CNVnator (Abyzov *et al.* 2011), CANVAS (Roller *et al.* 2016) and Control-FREEC (Boeva *et al.* 2012), short-read NGS-based analysis has had a limited impact on the detection of structural rearrangements, especially those present in a single individual (Sedlazeck *et al.* 2018a).

Many of the issues affecting short-read NGS-based detection of breakpoints are overcome by using long-read sequencing data (Oehler *et al.* 2023, Showpnil *et al.* 2024). Using standard library preparation protocols, these datasets consist of reads with a median size of 10 to 30 kb and a typical read depth for the human genome ranging from 20 to 30x. While the quality of the base calling is typically lower than that achieved by short-read sequencing technologies, their long lengths allow reads to be mapped with high confidence across low-complexity and repetitive sequences. Consequently, unlike short-read data, the breakpoints of large structural rearrangements can easily be identified by detecting multiple instances of reads whose individual alignments map, with high confidence, to the same two distinct regions of the genome. While a single read mapping to discontinuous regions may be an artefact of library production, when several reads show the same alignment pattern, it becomes highly suggestive of a genuine breakpoint event.

Several long-read alignment programs have been developed, such as Minimap2 (Li 2018), NGMLR (Sedlazeck *et al.* 2018b), and Ira (Ren and Chaisson 2021). When aligning reads that span a breakpoint, these aligners typically split the alignment into two parts. These split reads may be reported as either two independent alignments or as a single alignment interrupted by a large insertion or deletion that is described in the read’s alignment CIGAR string. For example, when reporting a split-read alignment, Minimap2 reports each alignment separately, labelling one as the primary alignment and the other as a secondary alignment, but also describes the other alignment within the alignment’s ‘tag’ data section. As a result of the increased use of long-read data, several long-read variant callers have been created for PacBio and ONT long-read data, with some, such as PBHoney (English *et al.* 2014), Sniffles v2 (Smolka *et al.* 2024), nanoSV (Cretu Stancu *et al.* 2017), and SVIM (Heller and Vingron 2019), aimed specifically at breakpoint detection rather than SNV detection. Once identified, these applications examine the orientation and alignment of reads spanning each breakpoint to determine both the type of rearrangement and then annotate it. However, due to the noisy nature of long-read base calling, the proposed annotation may be inaccurate but tends to be sufficiently close to allow the design of a PCR assay for both Sanger sequencing to determine the exact breakpoint validation and the cascade testing of the patient’s relatives.

Given the size and complex nature of the human genome, it is often necessary to stringently filter whole genome variant datasets that have been created by an automated pipeline to reduce the reporting of false positive findings. However, this filtering may reject genuine findings that occur in regions that are difficult to sequence and/or align data to or just happen to have a lower than expected read depth in the sample of interest. Consequently, this approach is reasonable when analyzing a large cohort of samples to detect common variants, but it may not be suitable when screening individual samples for patient-specific, novel disease-causing rearrangements.

Many established breakpoint detection tools, such as Sniffles2 (Smolka *et al.* 2024), SVJedi-graph (Romain and Lemaitre 2023) and cuteSV (Jiang *et al.* 2020) operate as command-line applications and offer no variant visualization options. Consequently, it is often necessary to visualize the data to ascertain if the annotation is correct. A number of desktop applications have been developed for alignment visualization, such as IGV (Thorvaldsdóttir *et al.* 2013), Ribbon (Nattestad *et al.* 2021), UCSC Genome Browser (Navarro Gonzalez *et al.* 2021), Tablet (Milne *et al.* 2016), Savant Genome Browser v2 (Fiume *et al.* 2012), and jBrowse2 (Diesh *et al.* 2023). As many of these applications were designed to display short-read data mapping to a specific region, they are often less than ideal for rearrangements that span extended regions or affect two unconnected loci. Table 4, available as supplementary data at *Bioinformatics* online compares the features of a range of long-read variant callers and programs designed to visualize aligned long-read data.

Here we describe AgileStructure–a user-guided program for the analysis of aligned, long-read data for the detection, visualization, and annotation of genomic structural rearrangements. As the analysis is user-driven, it is expected the approximate location of the variant is known with the candidate region screened in blocks of up to 10 Mb. This positional data may be suggested by observation of a patient karyotype or by linking the patient’s phenotype to variants known to cause similar conditions. AgileStructure may also be used to interrogate ambiguous observations, identified by other software packages, that require verification before they can be reported with confidence. The software and a user guide are hosted on GitHub at: https://github.com/msjimc/AgileStructure. While the application is primarily designed for the Windows OS, it can run on a range of Linux, BSD, and macOS systems via the Wine application layer infrastructure (https://www.winehq.org/ 2025).

## 2 Materials and methods

Experimental read data was either obtained from the NCBI SRA data archive or from the authors of the cited studies. Synthetic long-read data was produced using MakeSVGenome (https://github.com/msjimc/MakeSVGenome), wherein human chromosomes 7 and 8 were altered to incorporate various structural rearrangements. The altered reference sequences were subsequently used to create faux 10 kb reads at 1 kb intervals with alternating orientations. The mouse (mm10) and human (hg19) reference sequences were obtained from the UCSC Genome Browser, while the gene and repeat annotation files were obtained using the UCSC Genome Browser’s Table viewer (Karolchik *et al.* 2004) as described in AgileStricture’s GitHub user guide. Reads were aligned to the relevant genome by Minimap2 using the -ax map-ont and—secondary=no parameters. The resultant SAM files were then converted to BAM files, sorted by genomic position, and indexed using Samtools (Li *et al.* 2009).

## 3 Results

### 3.1 Design and implementation

AgileStructure is a desktop application written in C# that, while targeting the Windows desktop environment, can also run on a wide range of POSIX-compliant systems, including macOS, Linux, and BSD, with the compatibility layer Wine (https://www.winehq.org/, 2025), in conjunction with the .NET 6 or later framework (installed via Wine).

AgileStructure was developed using data aligned by Minimap2, which reports split reads as multiple alignments, with each secondary alignment’s details referenced in the primary alignment’s tag section; as a result, data from other aligners may not be compatible. All of the required alignment data is extracted from the indexed BAM file’s header and alignment sections. While no additional data is required, it is possible to import and display the positions of genes and repetitive sequences within the selected region.

**Figure 1:**
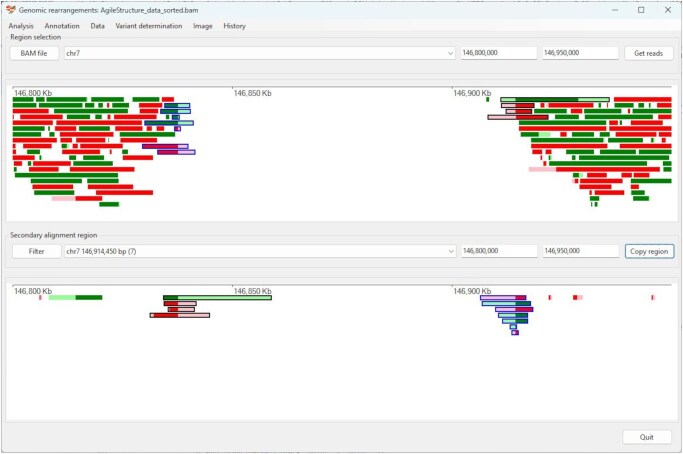
Read data mapping across a homozygous deletion in the CNTNAP2 gene. Dark green and red rectangles represent alignments to the forward and reverse strands, respectively. Pale green and red rectangles represent soft-clipped sequences in the mapped reads. The primary alignments are visualised in the upper panel, while the linked secondary alignments are shown in the lower panel. Split reads whose primary alignment is 5’ of the deletion are highlighted with a blue perimeter, while those on the 3 prime side have a blue perimeter.

The user interface consists of two distinct panels, each containing two text fields and a list box for the selection of a genomic region. The upper panel enables the selection of any region in the genome, displaying the primary alignments that map to it, while the lower panel displays their secondary alignments that map to a user-selected region (Fig. 1).

The genomic positions of the junctions between primary and secondary alignments of split reads shown in the upper panel are retained, with a putative breakpoint identified if 2 or more of these positions occur within 250 nucleotides. The breakpoint locations are used to limit the regions that can initially be viewed in the lower panel. Therefore, breakpoints are identified as a group of split reads whose primary alignments start or stop at the same point in the upper display and whose secondary alignments start or stop at the same point in the lower display. Initially, the lower panel will display reads mapping within 50 kb of a selected breakpoint; however, the size of this region can be adjusted to optimize the presentation of the rearrangement.

### 3.2 Distinguishing between simple and complex rearrangements

**Figure 2:**
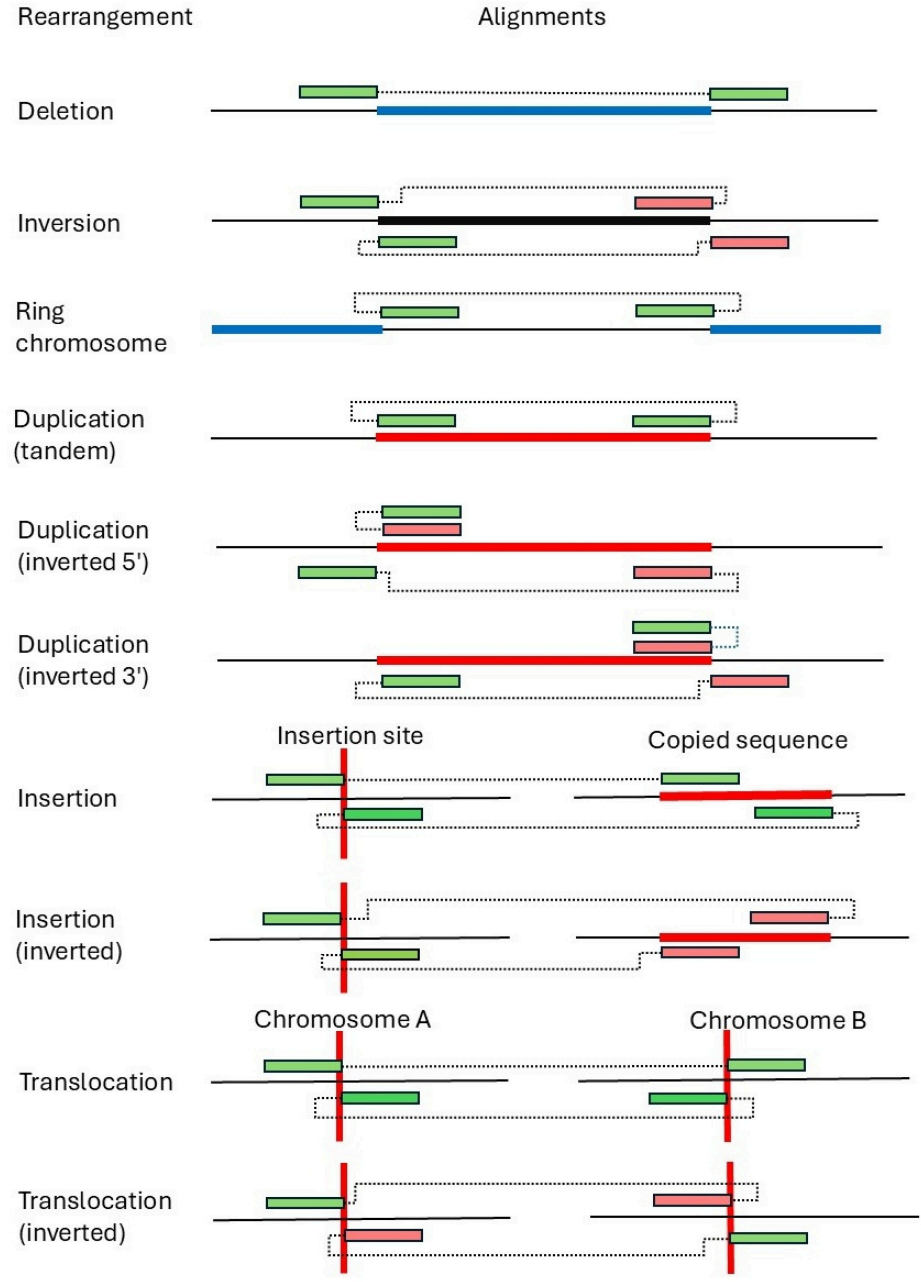
The arrangement of the primary and secondary alignments of split reads spanning a selection of structural rearrangements. The dotted line links contiguous sequences in the read with each primary and secondary alignment represented as a green or pink rectangle. If the linked primary and secondary alignments have different alignment orientations, the two rectangles are different colours. The affected sequence is shown as a thick blue (deleted), red (duplicated), or black (inverted) horizontal line as found in the reference genome, while the vertical red line shows the location of either an insertion or translocation as seen in the reference genome. For brevity, primary and secondary alignments are not indicated, but it should be noted that in this figure each read’s alignment will consist of one primary and one secondary alignment.

Many structural changes have a consistent copy number across the rearrangement and can be considered as simple rearrangements. Others, however, may have either deleted or duplicated sequences at the insertion site, creating a complex rearrangement where the copy number changes across the length of the variant. Simple rearrangements consist of two columns of split read alignments, except for insertions, which have three (Fig. 2), and their annotation is performed via the appropriate Variant determination submenu. When the split reads’ alignment pattern doesn’t match this profile, AgileStructure considers it to be a complex rearrangement, and their annotation is achieved via the Complex Rearrangement window.3

### 3.3 Simple rearrangements

When searching for a rearrangement at a poorly defined locus, the region, or part of it, is selected in the upper panel, and the locations of putative breakpoints are listed in the lower panel’s dropdown list control. These regions of secondary alignments are visualized in turn to identify true breakpoints. Once found, the split reads linked to the breakpoint are selected by clicking on them with the mouse, and the annotation is performed by selecting the relevant menu option in the Variant determination > Use soft clip data menu options. Since the alignment of reads may vary due to base calling errors, each split read is annotated in turn, with the resultant array of annotations used to create a single generic annotation. This is then displayed in a dialogue box that allows the user to save the relevant information to a file along with the sequence data of any selected read used.

**Figure 3:**
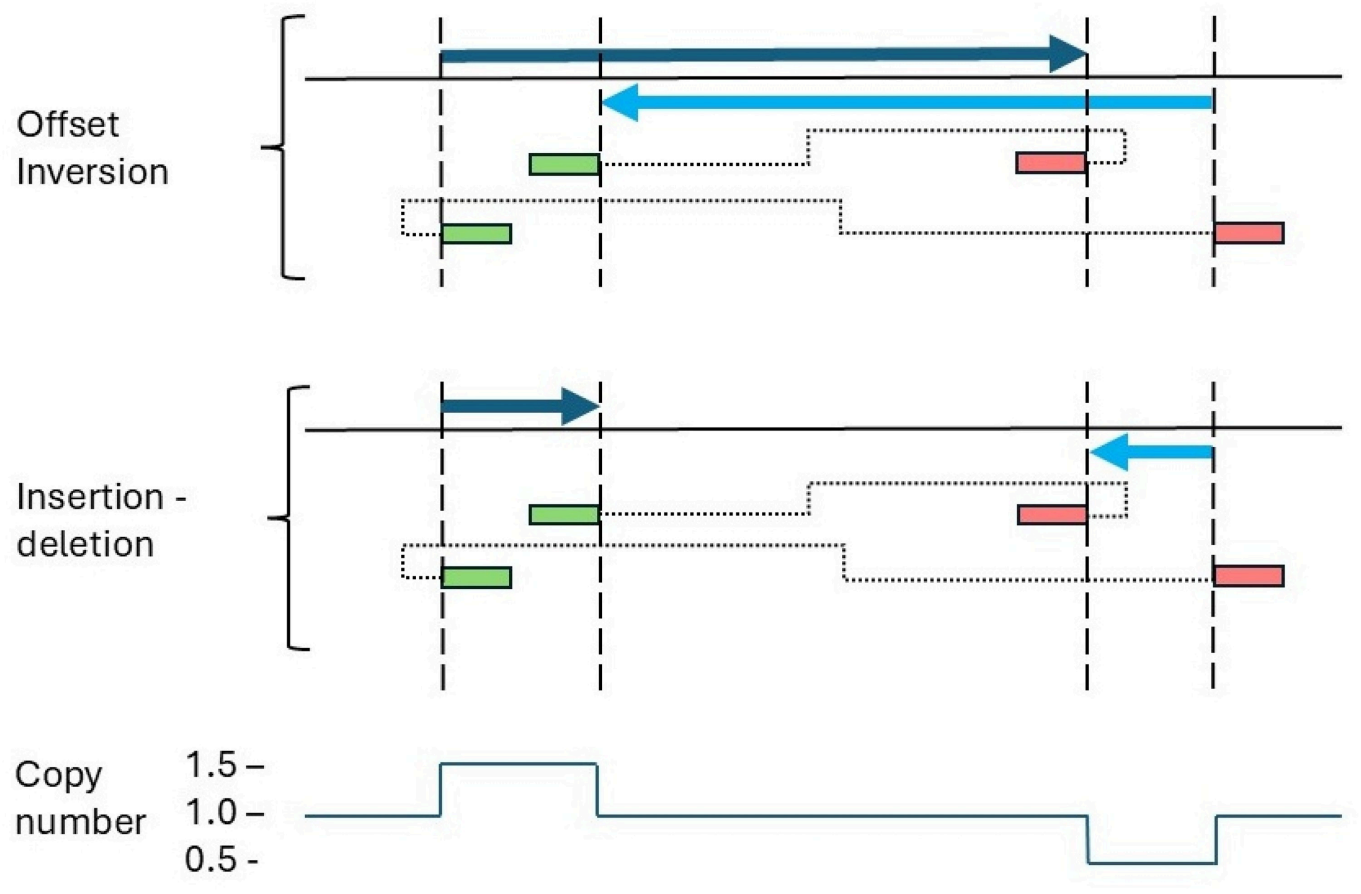
Offset inversions and insertion-deletions where the inserted sequence was inverted and inserted into the same chromosome as the original sequence could not be distinguished from each other. The dark and pale blue arrows identify the inserted sequence and the sequence deleted at the insertion site, respectively. The direction of the arrows indicates the orientation of the copied sequence. The green and pink rectangles represent the different alignments of split reads spanning a breakpoint. The colour of the rectangles is unimportant other than to indicate that the linked alignments do not have the same orientation. The vertical dashed lines indicate the position of the breakpoints. The dotted lines indicate how the alignments are connected in the long read. The copy number graph shows the copy number across both types of rearrangements.

If the type of rearrangement is not known, selecting the Variant determination > Use soft clip data > Variant type menu option will prompt AgileStructure to scan the alignments of the selected split reads to determine their orientation and position relative to the putative breakpoint. These alignment patterns are then compared to those expected for each of the different types of rearrangements as shown in Fig. 2 and described in the ‘Rearrangement annotation using split reads’ section of the Supplementary data (Figs. 1 to 6, Table 1, available as supplementary data at *Bioinformatics* online) to determine the most likely type of rearrangement.

### 3.4 Complex rearrangements

Usually, complex rearrangements present as either 3 or 4 columns of split-read alignments depending on whether one or both sides of the rearrangement are complex (see Fig. 8, available as supplementary data at *Bioinformatics* online and associated text), and their correct annotation requires the use of the Complex rearrangement window to select the relevant split reads and prompt their annotation. While two columns of alignments can be selected, these should represent a single breakpoint at which sequences have been either deleted or duplicated secondary to the insertion of the transposed sequence. The user guide on GitHub (https://github.com/msjimc/AgileStructure/tree/master/guide) contains examples of read selection for 68 simple and complex rearrangements created by MakeSVgenome. For most cases, AgileStructure can define the variant, but in cases where the annotation is ambiguous, it may be necessary to re-examine the reads selected. However, there are two situations where observation of long-read data cannot produce an unambiguous annotation. These are when differentiating between ring chromosomes and tandem duplications or distinguishing between an offset inversion and an insertion of an inverted sequence on the same chromosome as the original sequence, with loss of sequence at the insertion site. While the annotation of simple rearrangements is straightforward and may be completed with only a subset of the split reads, complex rearrangement analysis requires the selection of as many split reads as possible since the analysis compares the positions of alignments and the proportion of reads conforming to each alignment pattern.

### 3.5 Insertion and deletion identification using gapped reads

While primarily designed for the analysis of split reads as reported by aligners such as Minimap2, AgileStructure can also identify insertions and deletions encoded in the read’s CIGAR string such as lra. Rather than report a read spanning a structural rearrangement as two linked alignments, some aligners report them as a single gapped alignment with the rearrangement suggested in the CIGAR string. To use the CIGAR string, it must be possible to describe the global alignment as a series of consecutive local alignments who order reflect their order in the read’s sequence; consequently, gapped alignments can only be used to identify insertions and deletions. To simplify the data display, by default, insertions or deletions described in the CIGAR string are not shown. However, features larger than 10 bp can be displayed by selecting the Look for indels within a read menu option (Figs. 7a and 7b, available as supplementary data at *Bioinformatics* online).

As with the split alignments, the location and length of indels vary slightly between different reads due to poor base calling and/or difficulties aligning the sequences. For instance, the start and end positions of the deletion reported in Figs. 7a and 7b, available as supplementary data at *Bioinformatics* online vary by 182 bp and 187 bp, respectively, while the insert length of approximately 38 440 bp varied by only 8 bp. Consequently, when annotating a rearrangement, AgileStructure identifies the reported start and end points in each selected read and uses the median reported values in the reported annotation of the variant.

## 4 Validation

AgileStructure was used to analyse both synthetic and experimental data to demonstrate the accuracy of its annotations and utility in a research environment.

### 4.1 Synthetic data

A variety of *in silico* long-read datasets were generated using MakeSVGenome that consisted of reads derived from modified chromosomal sequences that contained either one of 32 simple rearrangements (deletions, insertions, inversions, duplications, translocations and a ring chromosome) or one of 36 complex insertion-deletion rearrangements. These could be an insertion where the sequence adjacent to the insertion was deleted or a duplication where the duplicated sequence was inserted either close to or within the copied sequence (Table 2, available as supplementary data at *Bioinformatics* online). Data from synthetic translocations were combined to produce alignments with both balanced and unbalanced translocations. Of the 68 different in silico rearrangements, ambiguous annotations occurred in only two scenarios:

#### 4.1.1 Tandem duplications and ring chromosomes

Despite their significant differences, ring chromosomes and tandem duplications present with the identical primary and secondary alignment patterns and are therefore not readily distinguishable (Fig. 2). However, they could be differentiated by observation of the individual’s karyotype or the rearrangement’s copy number. For a ring chromosome, the region between the breakpoints will have a diploid copy number, while telomeric sequences would be either haploid or absent. In comparison, the copy number between a duplication’s breakpoints would be elevated when compared to the rest of the genome.

#### 4.1.2 Offset inversions and inverted insertion-deletions

Figure 3 highlights a scenario where a sequence is inverted and then inserted into the same chromosome as the original sequence, with the loss of sequence at the insertion site. In this instance, it’s not possible to determine if the insertion site is distinct from the location of the copied sequence (an insertion-deletion) or if the transposed sequence is integrated into the original sequence’s locus with partial loss of the original sequence (offset inversion). If the distance between the breakpoints is large, observation of the patient’s karyotype may resolve the issue; if not, longer reads that span two columns of split reads may be required.

### 4.2 Experimental data

To demonstrate its real-world utility, AgileStructure was used to annotate a range of known rearrangements in datasets that had previously been reported as described below, with the reported variants summaries in Table 3, available as supplementary data at *Bioinformatics* online. As the *in silico* datasets contained read sequences directly copied from the reference sequence, their alignments were very clean. However, analysis of the experimental data revealed a significant number of possible ligation and base-calling errors. This resulted in a noisy display that could mask the columns of split reads in the primary alignment panel; however, when used in tandem with the read’s secondary alignments displayed in the lower panel, the structural rearrangements could easily be visualized.

### 4.3 Exemplars

*Deletion*: Watson *et al.* (2014) described a patient with a homozygous deletion in CNTNAP2. Sanger sequencing of the patient identified the deletion as the loss of chromosome 7, 146 534 699 to 146 611 541 (human reference genome, build hg19). AgileStructure annotated the rearrangement as chr7.146 534 703_146 611 542del. The 5' and 3' ends of the deletion were misplaced by +4 bp and +1 bp, respectively (Fig. 1).

*Inversion*: Watson *et al.* (2016) described a patient with a heterozygous pericentric inversion of chromosome 7 with the breakpoints, identified by Sanger sequencing, at positions 27 762 423 and 93 599 530 (human reference genome, build hg19). AgileStructure was able to annotate the rearrangement using reads with primary alignments at either chr7:27 762 423 or chr7:93 599 530. Using primary alignments mapped to chr7:27 762 423, the rearrangement was annotated to be chr7:27 762 427_93 599 530inv, while using primary alignments mapped to chr7:93 599 530, the rearrangement was annotated to be chr7:27 762 425_93 599 531inv. The reported breakpoints were 4 bp or less from the published location.

*Translocations*: Hu *et al.* (2019) described a patient with a translocation between chromosomes 6 and 8 (NCBI SRA sample: SRR9982132). The published rearrangement was t(chr6; chr8) (g.167 281 717: g.113 696 089) (human reference genome, build hg19), with AgileStructure identifying the variant as t(chr6; chr8) (g.167 281 716; g.113 696 100) using primary alignments on chromosome 6 and t(chr6; chr8) (g.167 281 719; g.113 696 098) using primary alignments mapping to chromosome 8. The annotations produced by AgileStructure for the chromosome 6 breakpoint were either −1 or +2 bp from the reported position, while the breakpoint on chromosome 8 was either +11 or + 9 bp from the published position.

*Duplication*: Sailer *et al.* (2021) describe a transgenic Agmo knockout mouse (NCBI SRA sample: SRR12783028) that, rather than having the start of the Agmo gene deleted as intended, had a tandem duplication of the 5' end of the Agmo gene, which was described as chr12:37 206,133–37 300,425dup (mouse reference genome, build mm10). Analysis of the long-read data by AgileStructure identified the duplication to be chr12:37 206,133_37 300 424dup, which is in agreement with the published variant on the 5' side and out by −1 bp on the 3' side.

## 5 Discussion

AgileStructure is a simple-to-use application for the guided annotation of structural rearrangements. To demonstrate its ability to correctly identify a wide range of rearrangements, synthetic long-read data sets with known rearrangements were created. The program’s usability was demonstrated using experimental long-read data for which the rearrangement had previously been published. Variant analysis was divided into two workflows: a simple rearrangement analysis pathway and a complex rearrangement pathway. A rearrangement is considered simple if it can easily be described as a deletion, insertion, inversion, duplication, translocation, or ring chromosome and generally has a consistent copy number across the rearrangement. The remainder were considered complex and could be described as an insertion-deletion event where a sequence is deleted at the insertion site. The copy number across these rearrangements may vary. Whether a rearrangement is simple or complex can typically be determined by observation of the location of the split reads delimiting the variant: simple rearrangements (except insertions) have 2 columns of split reads, while complex rearrangements have 3 or 4. Fig. 8, available as supplementary data at *Bioinformatics* online shows the changes in the location of a split-read’s alignments as the size of the deleted/duplicated sequences and the split-read alignments shift.

While robust, this classification is not perfect; for instance, an unbalanced translocation is considered a simple rearrangement by AgileStructure but does not have a consistent copy number. Irrespective of whether the unbalanced translocation occurred because a patient inherited one derivative chromosome from a parent with a balanced translocation or because the exchange of telomere sequences was not clean, each translocation breakpoint should be analyzed using the simple rearrangement analysis pathway. The classification also omits very complex rearrangements where multiple events have occurred at the same locus. This may occur in cancers with highly unstable genomes or in patients whose rearrangement occurred within a preexisting polymorphic structural rearrangement that was not present in the reference sequence. In these situations, the rearrangement may need to be manually processed.

Simple rearrangements can generally be annotated using reads selected from just one column of split-read alignments, except for inverted duplications and insertions, which only require reads aligned to both sides of the insertion site. However, since the columns of split reads aligning to either side of a breakpoint may present as two distinct groupings (Fig. 8, available as supplementary data at *Bioinformatics* online), the annotation of a complex rearrangement requires the selection of two columns of split reads, each representing one side of a single breakpoint. Consequently, the analysis of complex rearrangements is more involved, and their annotation is performed using the Complex rearrangement window, which guides the user through the alignment selection.

Long-read sequence data can be used to unambiguously annotate most simple rearrangements, complex rearrangements that involve two chromosomes, or when the transposed sequence is not inverted. However, long-read sequencing data cannot unambiguously differentiate between ring chromosomes and tandem duplications, and, as Fig. 3 demonstrates, an inverted insertion on the same chromosome as the copied sequence cannot be resolved from an offset inversion. While copy number analysis can differentiate between a ring chromosome and a tandem duplication, it cannot distinguish between an inverted insertion and an offset inversion. Ultimately, these may only be resolved by direct observation of the patient’s chromosomes or by resequencing the sample with a greater read length that would span a proposed feature.

The detection and annotation of rearrangements may also fail if the aligner cannot unambiguously align the read. This may be due to either the transposed sequence or the insertion site’s sequence not being present in the reference sequence used by the aligner. This could arise if a virus whose sequence was not included in the reference genome was integrated into the genome or the rearrangement involved unsequenced regions of the genome, such as the p arm of an acrocentric chromosome. A similar scenario may occur if the breakpoint occurred in a highly polymorphic extended region of low complexity and/or repetitive sequence. In this situation, the base calling errors associated with each read may lead to significant variation in how the reads are aligned, leading to the break point being disregarded as no consistent pattern of alignment was achieved.

AgileStructure differs significantly from many of the currently available software packages for the annotation of rearrangements using long-read data in that its analysis is user-driven and requires prior information of the rearrangement’s location and type. While this may appear to be a significant limitation, many complex rearrangements can only be correctly annotated when alignments from more than one location are simultaneously processed. Annotation of each group of split reads of a complex rearrangement independently may lead to a failure to generate a correct annotation. For instance, the selection of one column of split reads from a complex insertion-deletion involving two chromosomes may be annotated as a translocation.

Consequently, AgileStructure is not intended to form part of an automated variant detection pipeline such as those incorporating Sniffles2 or SVJedi graph. Instead, it is designed to visualize long read alignments across a known disease locus or a region previously implicated by a single heterozygous pathogenic variant or highlighted by a low confidence structural variant call from an automated workflow. AgileStructure can then determine the type of structural variant present and define its breakpoint coordinates with sufficient accuracy to support the design of a PCR based confirmatory assay.

Many simple rearrangements can be easily annotated using the Human Genome Variation Society (HGVS) nomenclature (Hart *et al.* 2024); however, the annotation of many complex rearrangements can be very difficult, leading to a number of different classification and annotation nomenclatures been proposed (Taschner and den Dunnen 2011, Ordulu *et al.* 2014). Unfortunately, their use can be subjective; for example, if DNA is deleted at the site of the insertion of an inverted sequence, the rearrangement could be described as an indel; however, as the distance between the insertion site and the originating sequence reduces, it would become annotated as an inverted duplication. If the insertion site is within the copied sequence and the deleted sequence was large, it may be described as an inversion with duplicated or deleted flanking sequence(s): reaching the point at which the naming convention shifts is subjective and susceptible to misunderstanding. Consequently, AgileStructure reports complex rearrangements descriptively, stating the origin of the transposed sequence, whether it has been inverted relative to the sequence in the reference genome, and the location and extent of sequence loss at the insertion site. This enables all complex rearrangements to be reported in the same format, which can then be used to annotate the rearrangement in the user’s preferred format. Currently, AgileStructure is limited to variants that contain only the sequences flanking the breakpoints in the reference genome to which the reads are aligned. It is hoped that future versions will overcome this limitation. Similarly, we intend to extend support to additional aligners that report secondary alignments in the primary alignment’s tag section, as these aligners continue to rise in prominence.

In summary, we present AgileStructure, a desktop application that is designed specifically to aid the analysis of aligned long-read data for the localization, visualization and annotation of structural rearrangements whose presence has been suggested by previous analysis methodologies. While several command line applications have been developed to detect rearrangements in long-read sequencing data, their analysis may either be unduly stringent, rejecting the deleterious variant of interest, or too lax, exporting an extensive list of poor-quality artefactual variants. The visualization of long-read data carries a substantial processing cost. While this cost is negligible when analyzing a single locus, it becomes significant when attempting to process entire chromosomes or whole genomes. Because AgileStructure’s analysis is tightly coupled to its visualization of aligned reads and requires user input to select variants of interest, it is not suitable for whole genome analysis. However, AgileStructure is specifically designed to interpret and visualize data across a targeted region, making it well suited to researchers and clinical scientists who need to identify a specific deleterious variant in an individual sample rather than generate a comprehensive catalogue of rearrangements with high quality scores across a cohort.

## Supplementary Material

btag294_Supplementary_Data

## Data Availability

Source code, binaries, user guide, and example aligned read data, are available on GitHub: https://github.com/msjimc/AgileStructure. An archived version is also available on Zenodo at https://doi.org/10.5281/zenodo.18610110.
