# Supplementary material for: Utilization of long-read sequencing for the detection of structural rearrangements with AgileStructure
Source: Bioinformatics. 2026 May 8;42(5):btag294. doi: 10.1093/bioinformatics/btag294 (PMC13218792; doi:10.1093/bioinformatics/btag294)
Supplement: btag294_Supplementary_Data [file btag294_supplementary_data.docx]

# Supplementary Data

Description for figures 1 to 6. Dark green and red rectangles represent forward and reverse primary alignments in the upper panel and the second alignments in the lower panel. Pale green and red rectangles represent unaligned sequences in the mapped reads. The breakpoints are highlighted by blue and black arrows. For insertions, a purple arrow is used to indicate the breakpoint at the site at which the sequence is inserted. Figures are shown in pairs, with the first figure showing the display when all reads are displayed, while the following images show only the visualisation of split reads.

## Rearrangement annotation using split reads

### Deletions:

If an entire deletion is visualised, it appears as two columns of alignments in both the upper and lower panels. Each read’s primary and secondary alignments are always in the same orientation and occur outside of the deletion (supplementary 1a and 1b).


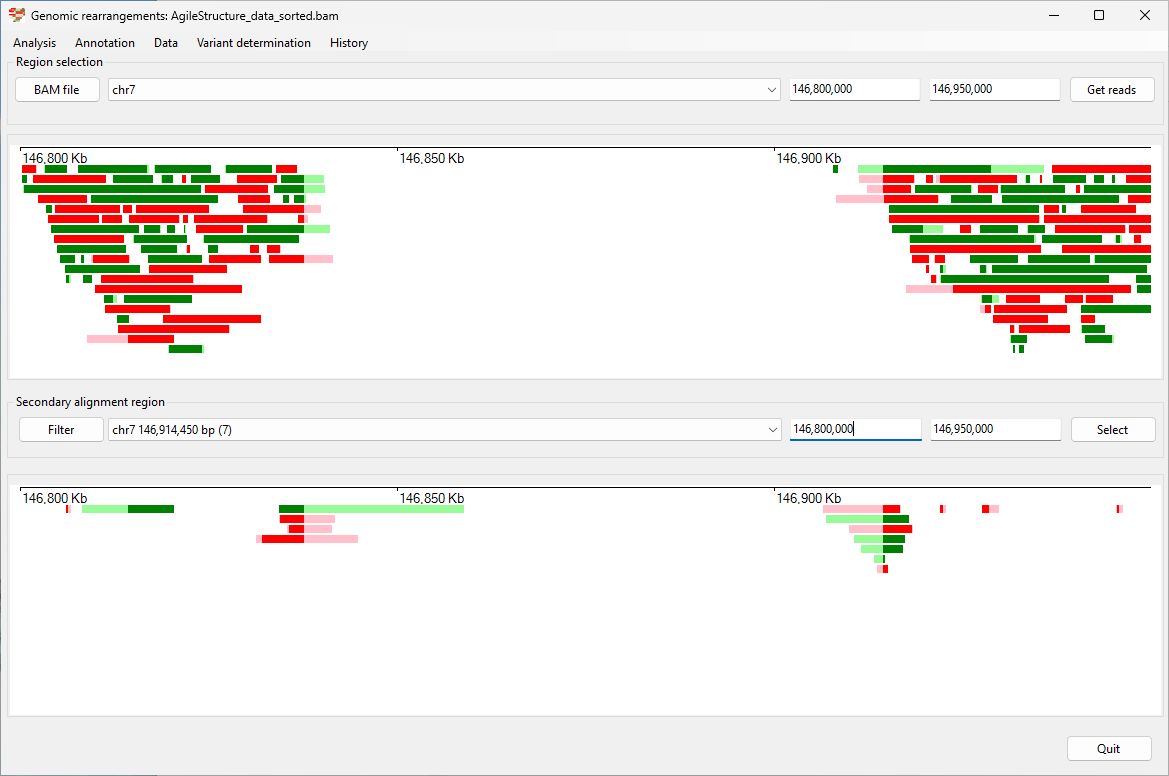


Figure 1a. The display of all reads mapping at a homozygous deletion


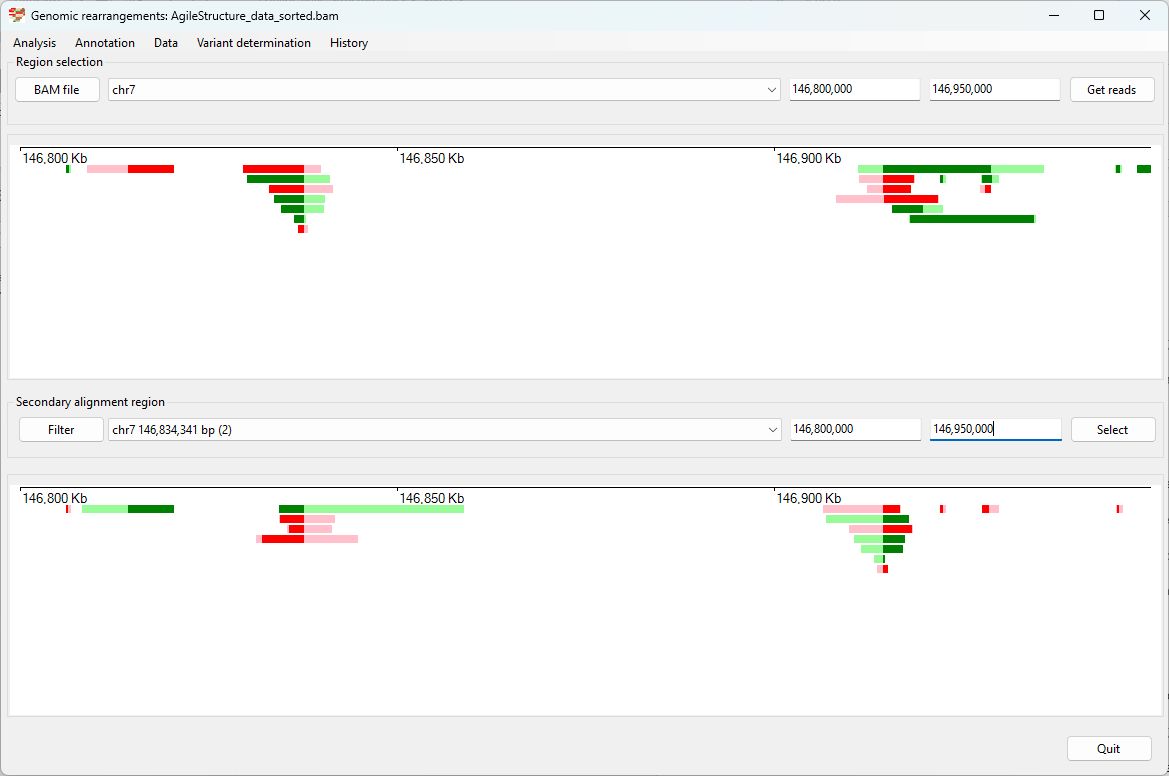


Figure 1b. The display of only split reads mapping at a homozygous deletion

### Inversions:

Like a deletion, when the entire inversion is selected, it appears as two columns of alignments in each panel, with primary alignments on one side of the inversion to the linked secondary alignments on the other side. However, unlike deletions, the orientation of the primary read is the opposite of that of its secondary alignment. Additionally, if a primary alignment maps outside the inversion, the secondary alignment will map within the inversion and vice versa. When the inversion is too large to be viewed in its entirety, it is possible to view each side of the inversion independently (supplementary Figures 2a to 2d).


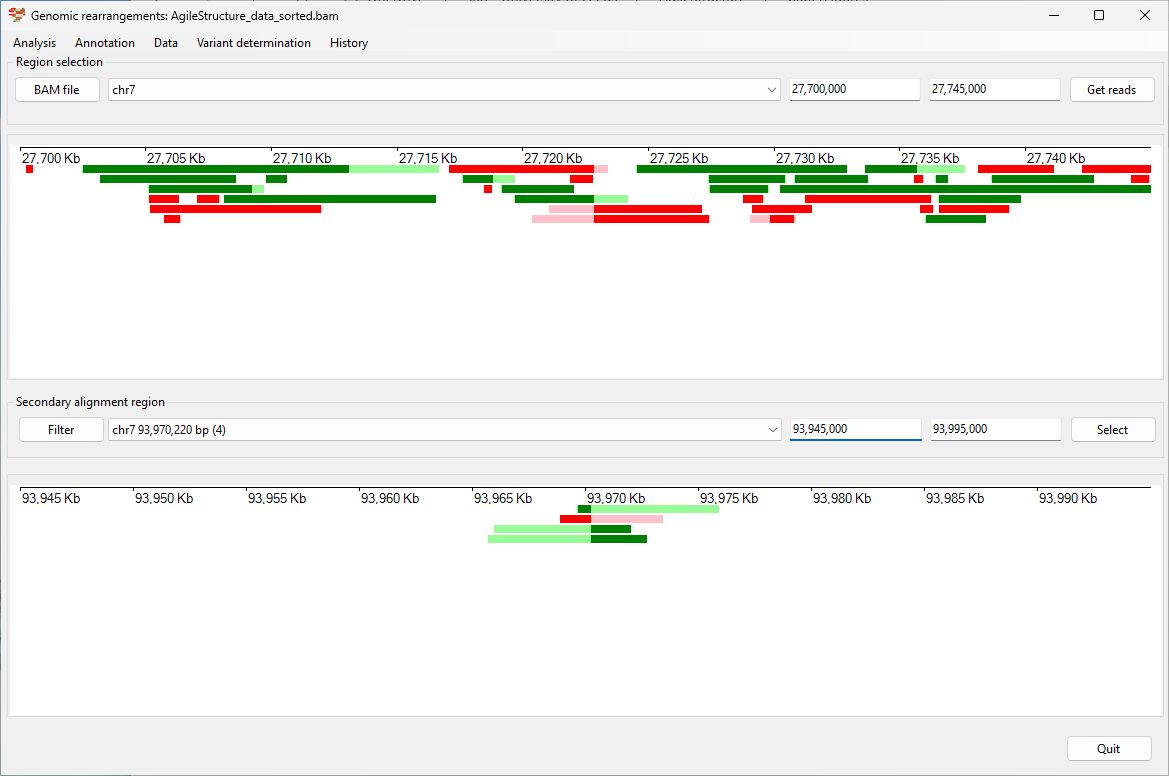


Figure 2a. The display of all reads mapping to the 5’ most breakpoint of an inversion


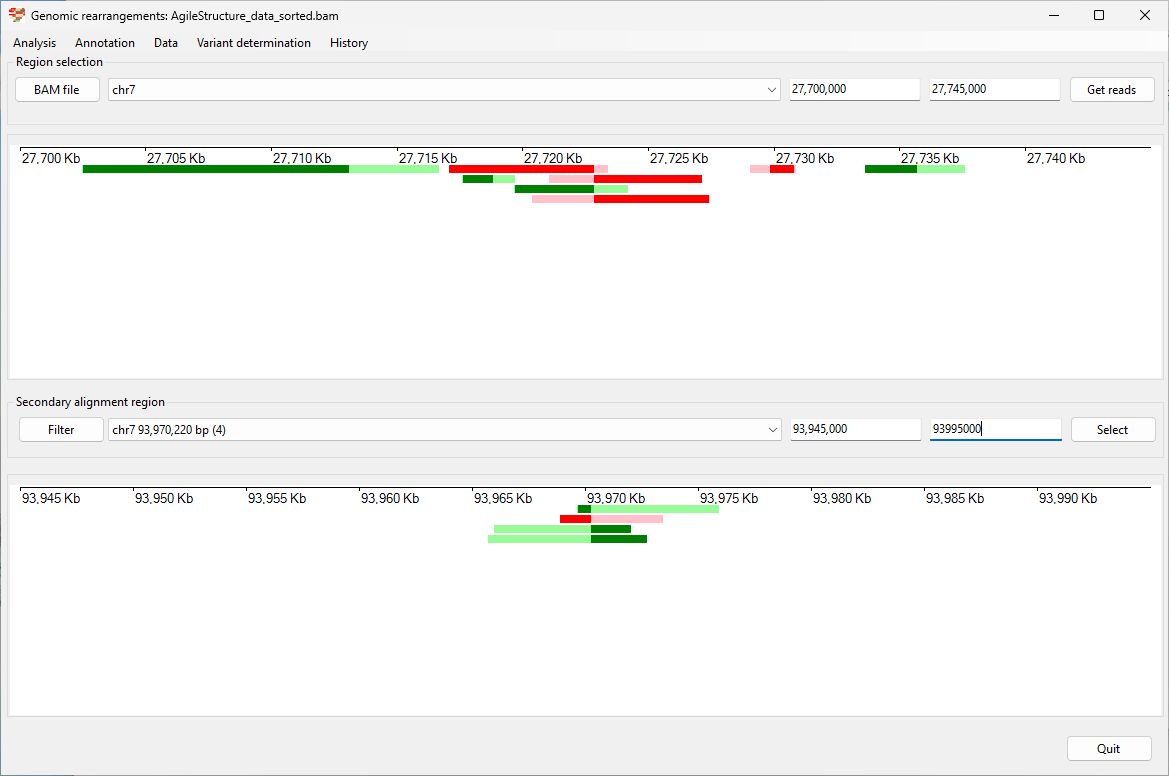


Figure 2b. The display of split reads mapping to the 5’ most breakpoint of an inversion


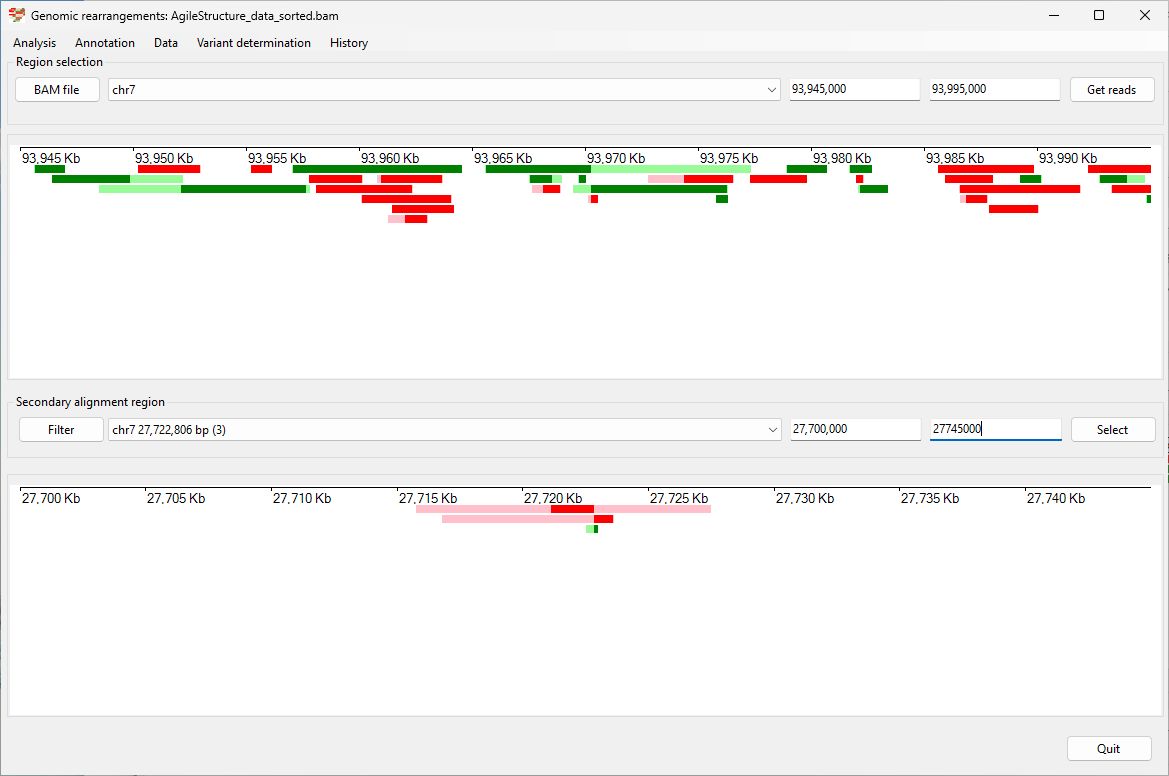


Figure 2c. The display of all reads mapping to the 3’ most breakpoint of an inversion


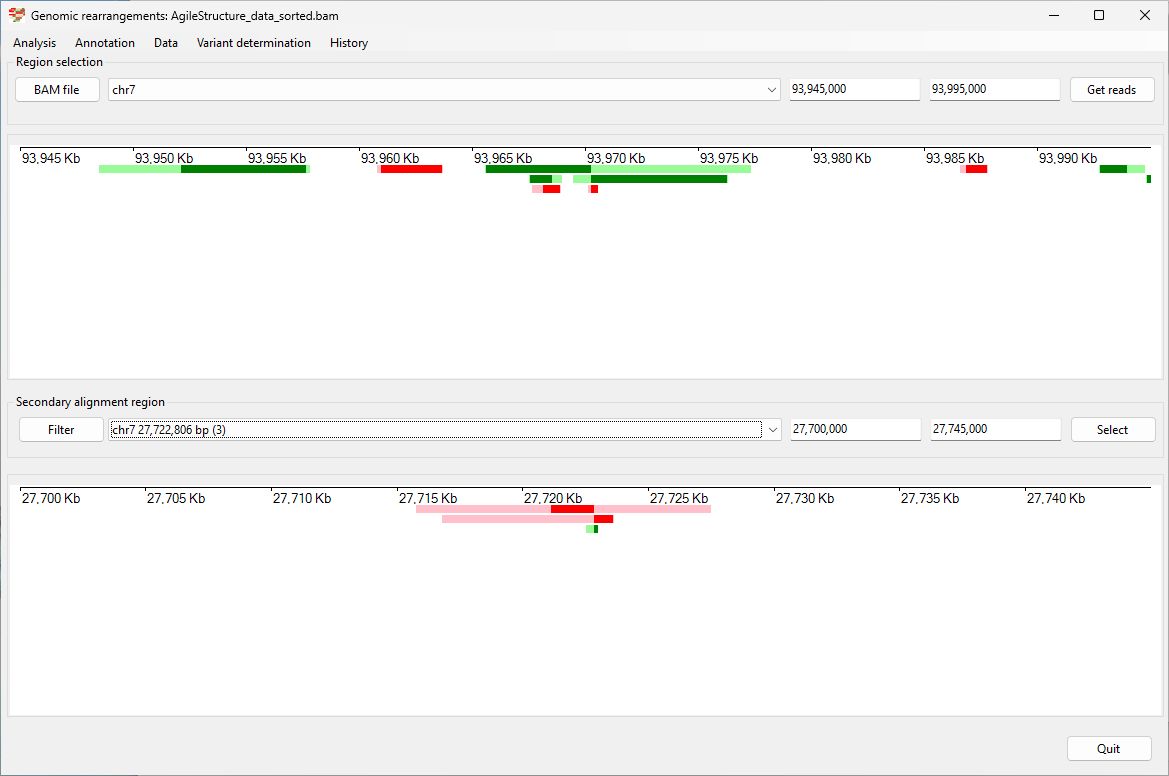


Figure 2d. The display of spit reads mapping to the 3’ most breakpoint of an inversion

### Duplications:

There are three types of duplications: tandem duplications, in which the inserted sequence is in the same orientation as the copied sequence, and inverted duplications with the transposed sequence inverted and inserted at either the 5’ or 3’ side of the copied sequence.

#### Tandem duplications:

Tandem duplications appear as two columns of alignments in both the upper and lower panels, with the orientation of a read’s primary alignment matching that of its secondary alignment. If a primary alignment occurs within the duplicated sequence, its linked secondary alignment lies outside the duplicated sequences and vice versa (supplementary Figures 3a and 3b). The alignment pattern for a tandem duplication is identical to that of a ring chromosome (see below).


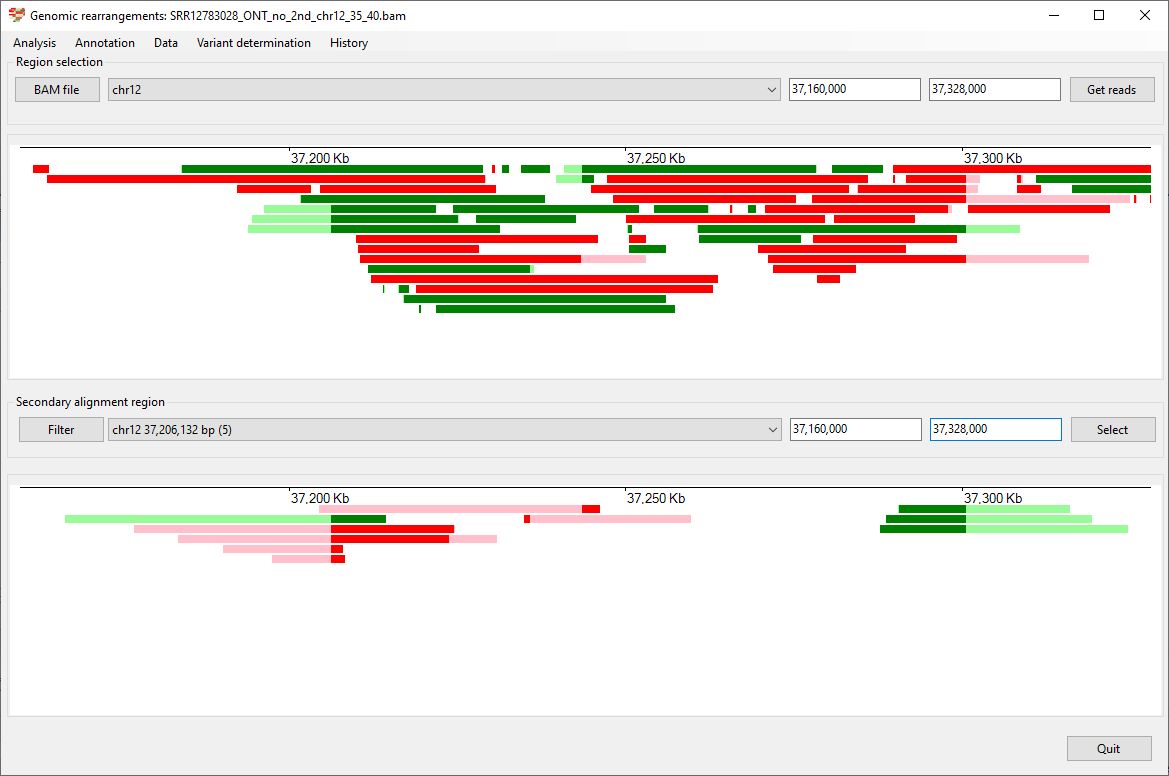


Figure 3a. The display of all reads mapping to the breakpoints in a tandem duplication


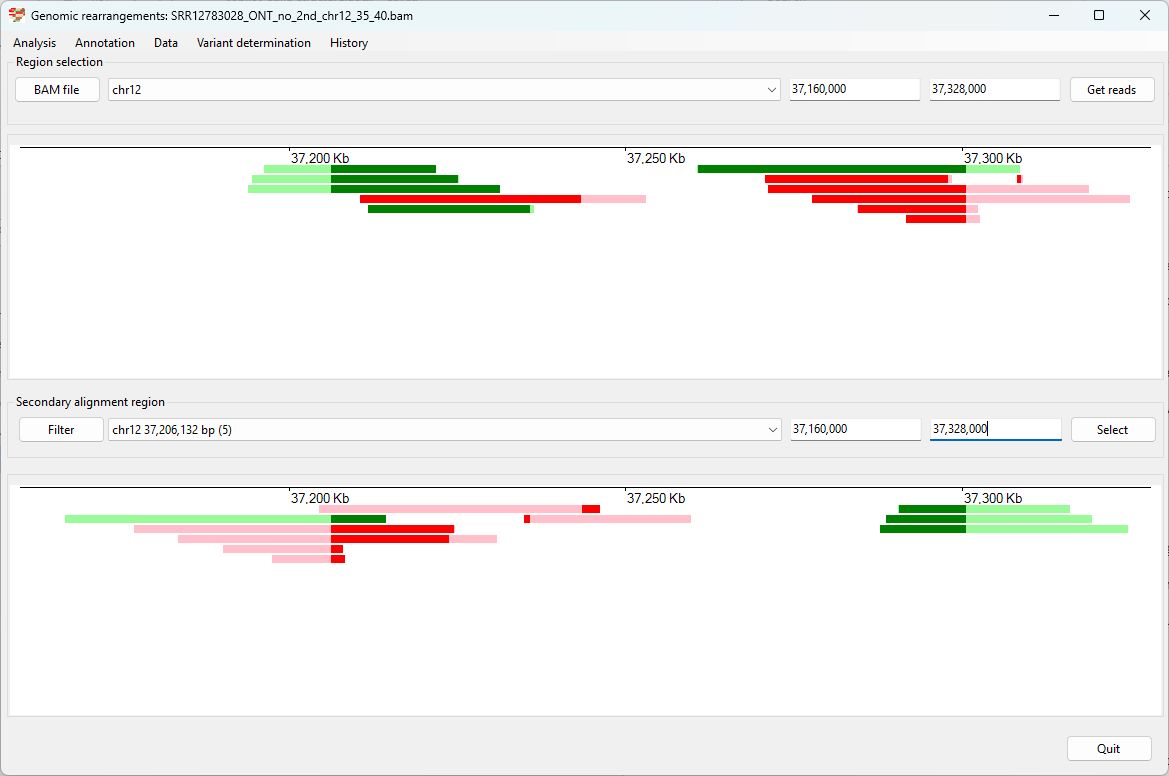


Figure 3b. The display of split reads mapping to the breakpoints in a tandem duplication

#### Inverted duplications:

Both types of inverted duplications consist of two columns of split reads that display three of four distinct alignment patterns. Half of the aligned split reads have a primary alignment at one side of the duplicated sequence and a secondary alignment at the other side, with either both alignments inside or outside the duplicated region. However, the remaining split reads will have both primary and secondary alignments at the same location. For inverted duplications with the inverted sequence inserted at the 5’ end of the original sequence, these reads will be located at the 5’ most position, and both alignments will occur within the duplication (see supplementary Figure 3c and 3d). For inverted duplications with the insertion at the 3’ end of the copied sequence, these alignments will occur at the 3’ end of the duplicated sequences, and one alignment will occur outside the copied region and the other within the duplicated region (see supplementary Figure 3e and 3f). Irrespective of the type of inverted duplication, the orientation of the secondary alignments will differ from the primary alignment’s orientation.


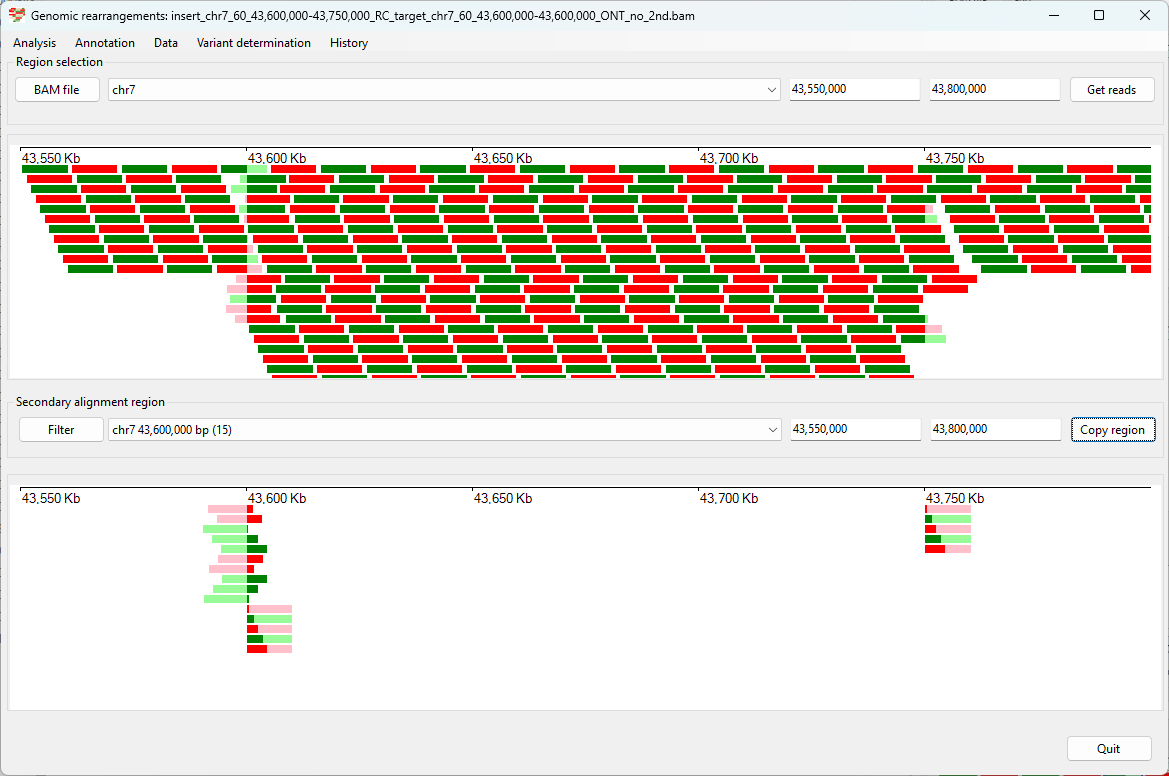


Figure 3c. The display of synthetic data in which all the reads mapping to the breakpoints in an inverted duplication, in which the inserted sequence is inserted at the 5’ end of the copied sequence (black arrow)


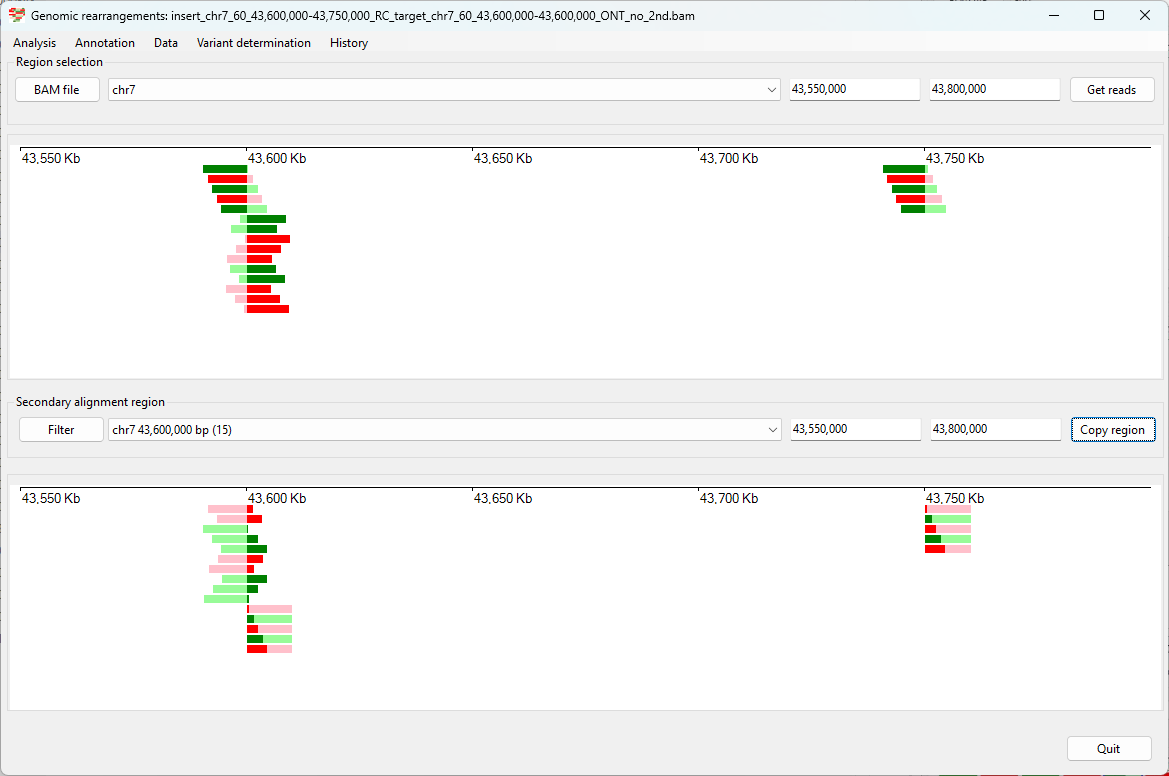


Figure 3d. The display of synthetic split reads mapping to the breakpoints in an inverted duplication, in which the inserted sequence is inserted at the 5’ end of the copied sequence (black arrow)


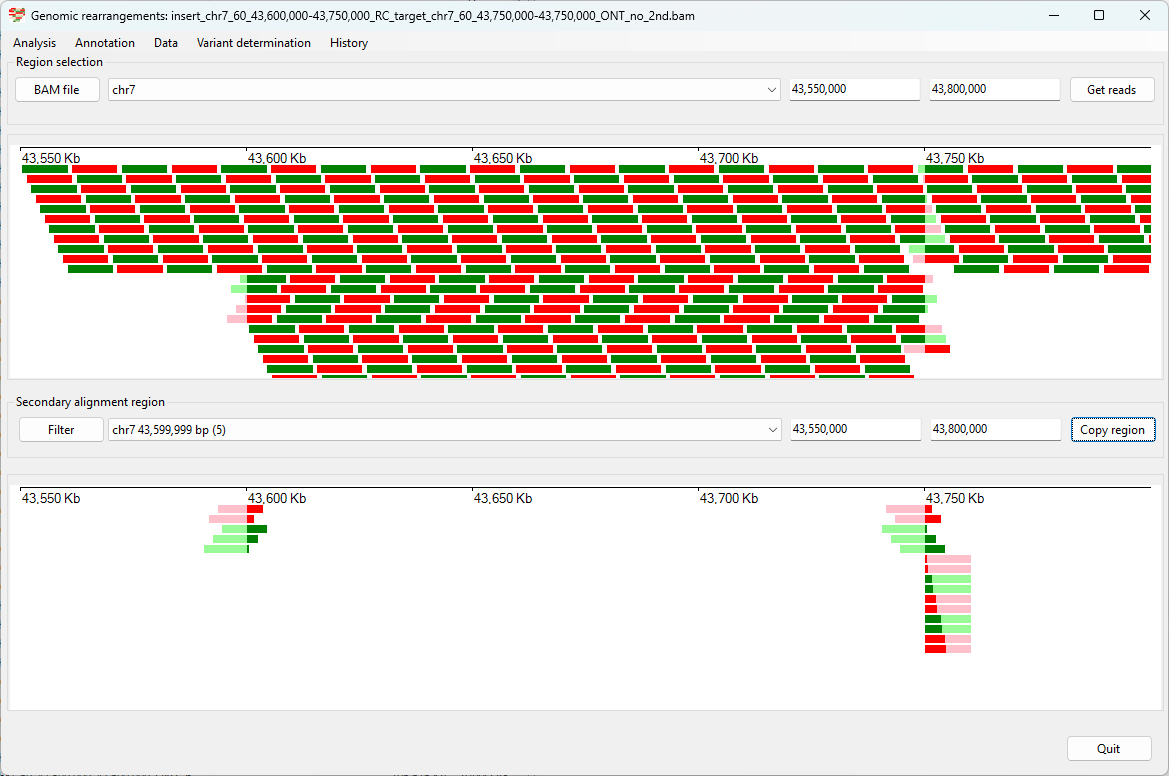


Figure 3e. The display of synthetic data in which all the reads mapping to the breakpoints in an inverted duplication, in which the inserted sequence is inserted at the 3’ end of the copied sequence (blue arrow)


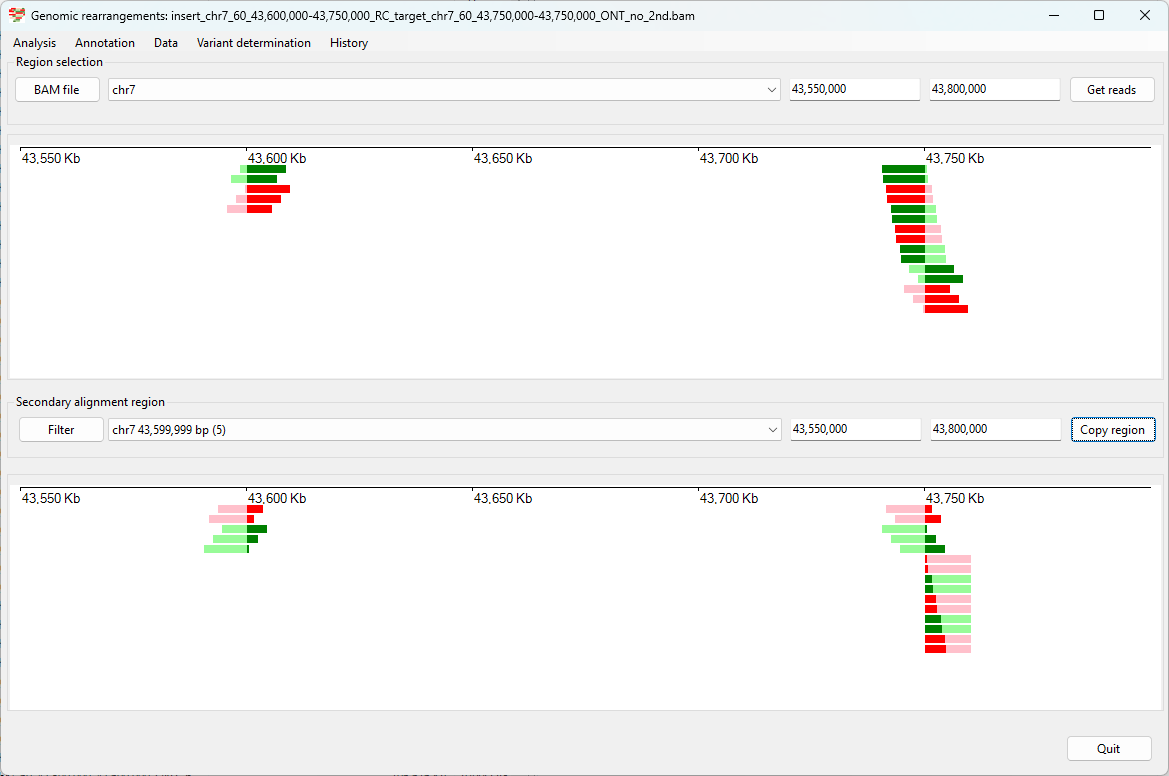


Figure 3f. The display of synthetic split reads mapping to the breakpoints in an inverted duplication, in which the inserted sequence is inserted at the 5’ end of the copied sequence (blue arrow)

### Insertions:

Unlike other simple rearrangements, insertions consist of three columns of reads: one identifies the point of insertion, while the other two delimit the original location of the copied DNA. If the inserted sequence is in the same orientation as the original sequence, all alignments on the 5’ side of the insertion site will have linked alignments on the 5’ side of the duplicated sequence, and alignments on the 3’ side of the insertion point will have linked alignments on the 3’ side of the duplicated sequence. Linked alignments will also share the same orientation. However, inverted insertions differ; those alignments on the 5’ side of the insertion point will have linked alignments at the 3’ end of the duplicated sequences, and alignments to the 3’ side of the insertion point are linked to alignments at the 5’ end of the duplicated sequence. Also, the orientation of the primary and secondary alignments is switched (see supplementary Figures 4a to 4d). To achieve this pattern, the inserted sequences must also be present in the alignment; consequently, if the inserted sequence originates from a virus or a transgenic construct, this sequence must also be present in the reference sequences used in the alignment.


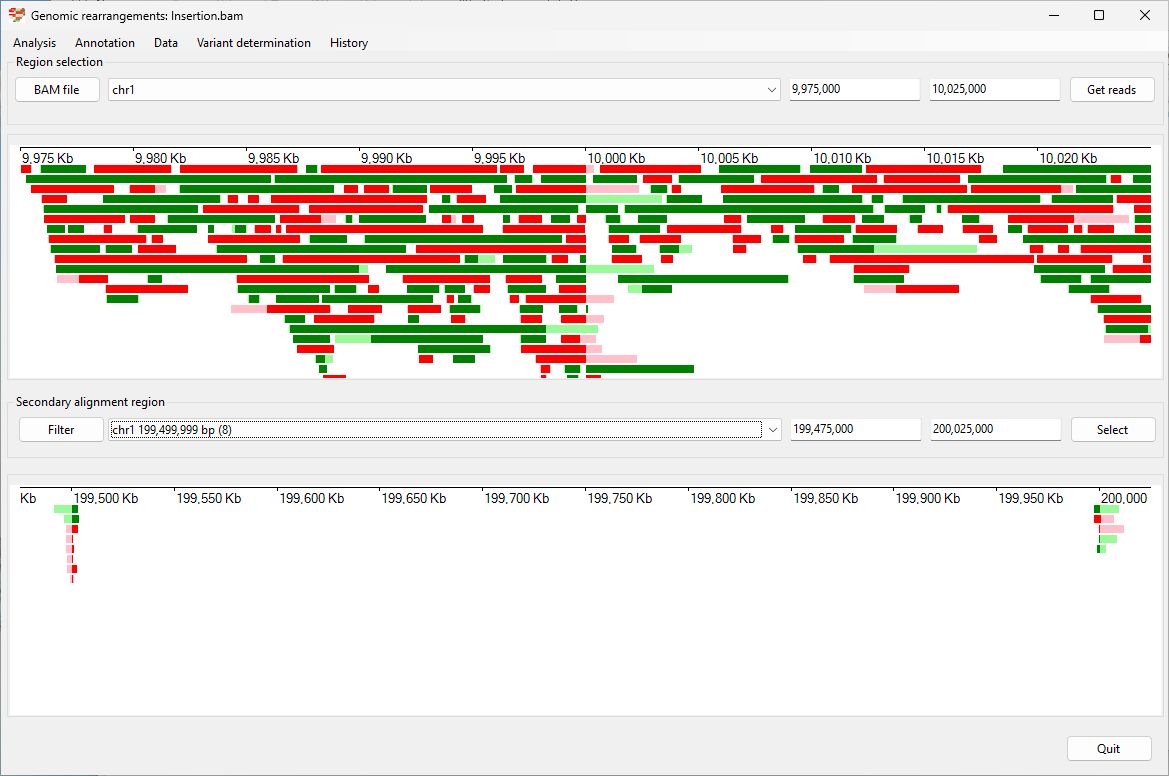


Figure 4a. The display of all reads mapping to the insertion point (upper panel) and the site of the copied sequence (lower panel) of an insertion


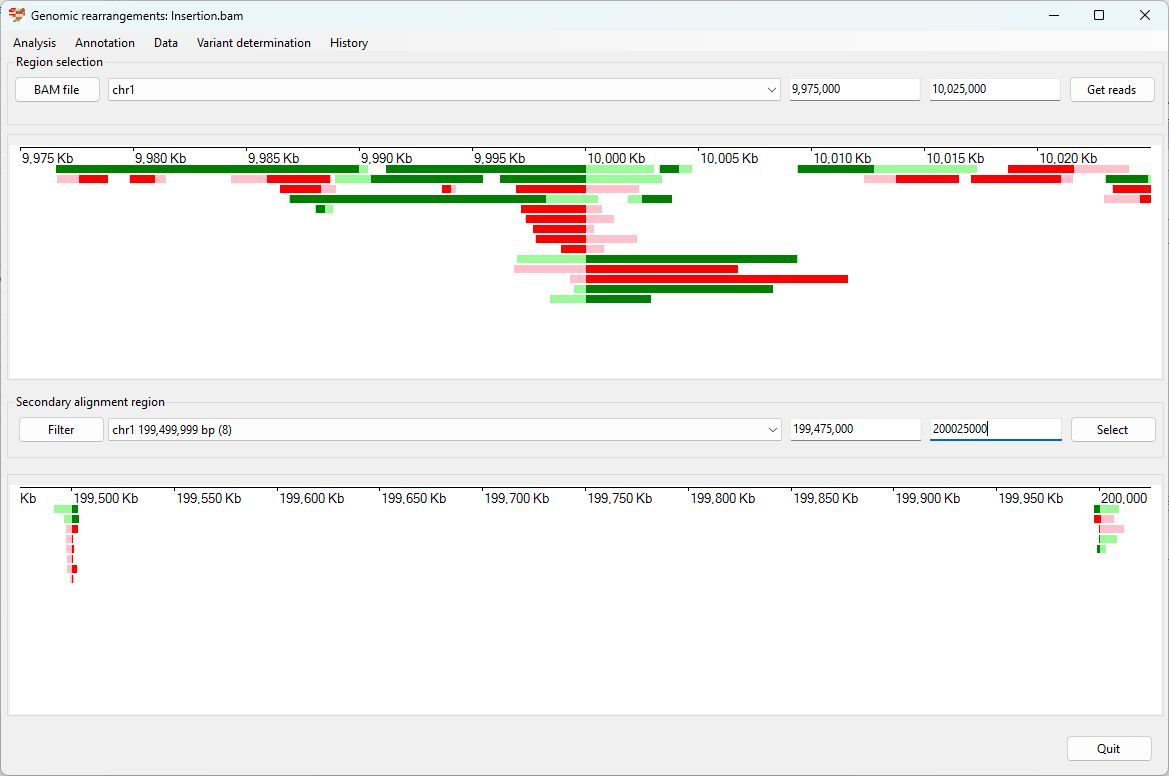


Figure 4b. The display of split reads mapping to the insertion point (upper panel) and the site of the copied sequence (lower panel) of an insertion


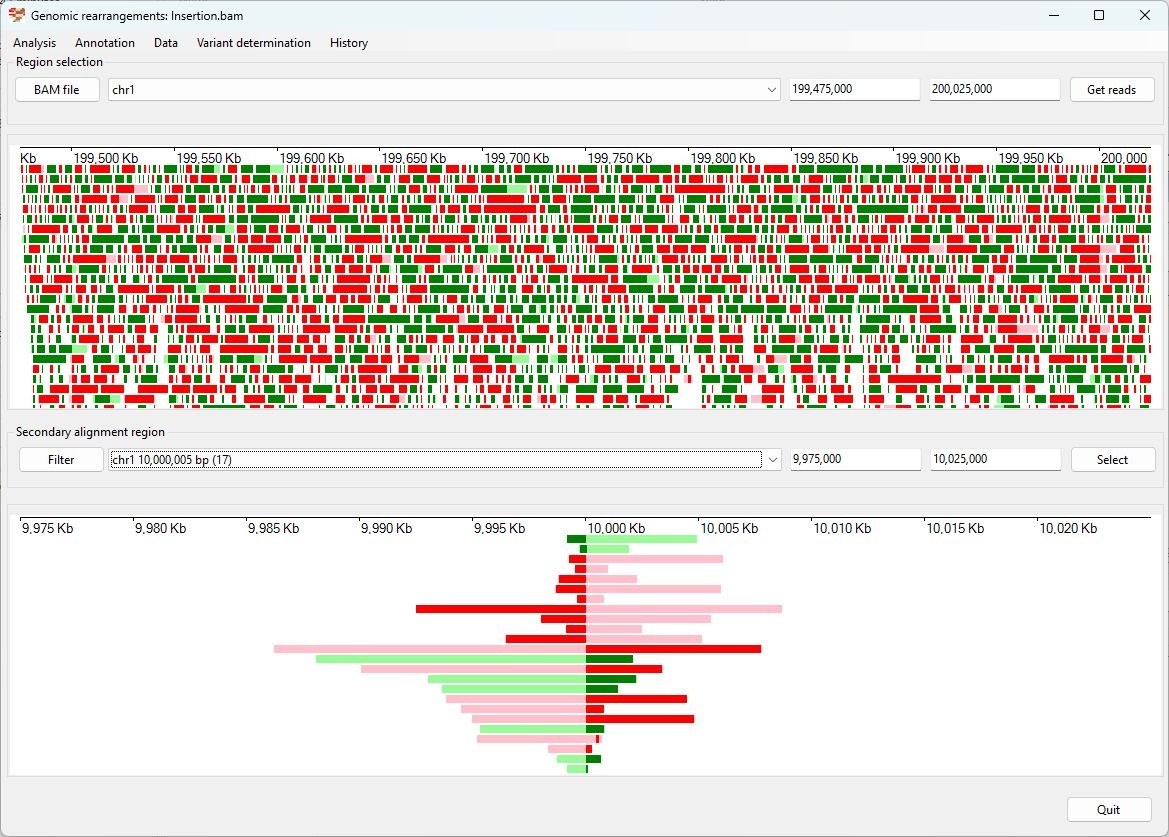


Figure 4c. The display of all reads mapping to the location of the copied sequence (upper panel) and the site of of its insertion (lower panel) of an insertion


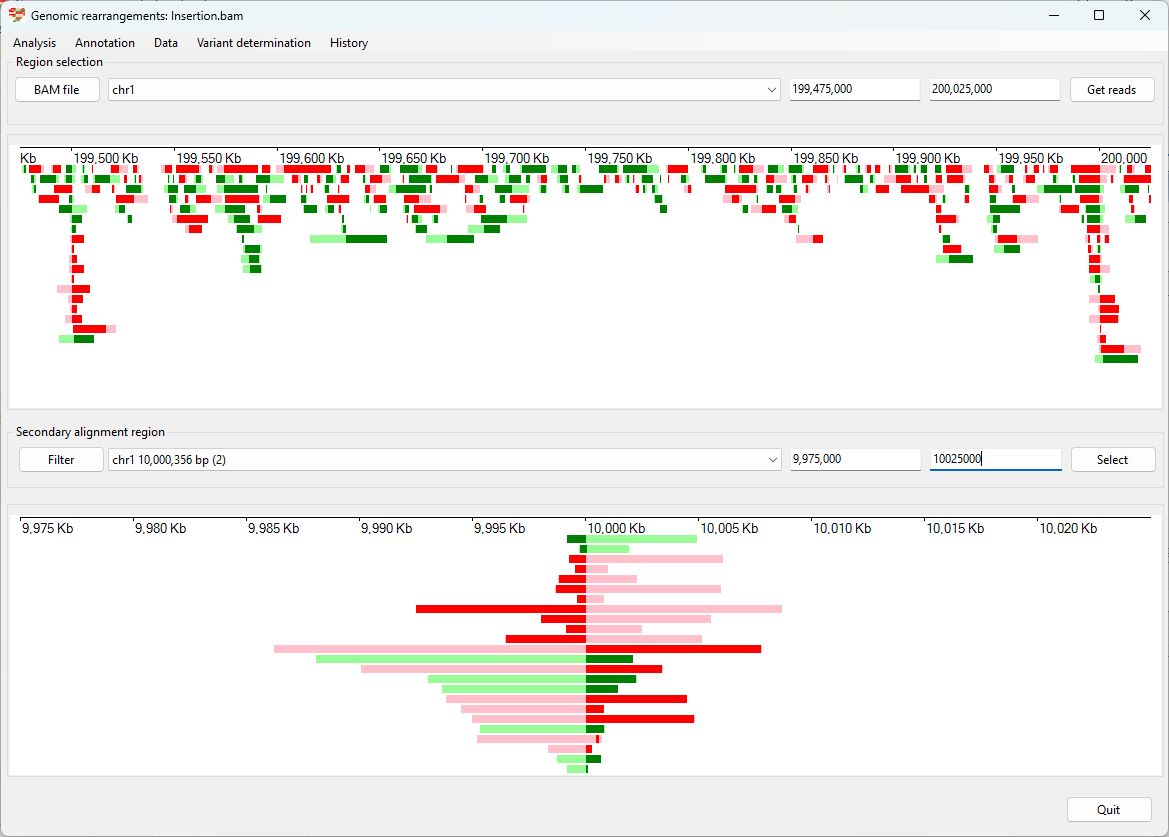


Figure 4d. The display of split reads mapping to the location of the copied sequence (upper panel) and the site of of its insertion (lower panel) of an insertion

### Translocations:

All translocations consist of a single column of split-read alignments in each of the panels, with the primary and secondary alignments mapping to different chromosomes. The orientations of a read’s primary and secondary alignments depend on the translocation and the orientation of the affected chromosomes in the reference sequence. However, all reads mapped to a specific translocation will exhibit the same pattern. If alignments are biased to one side of a breakpoint, AgileStructure will suggest that the translocation is not balanced (supplementary Figures 5a to 5d).


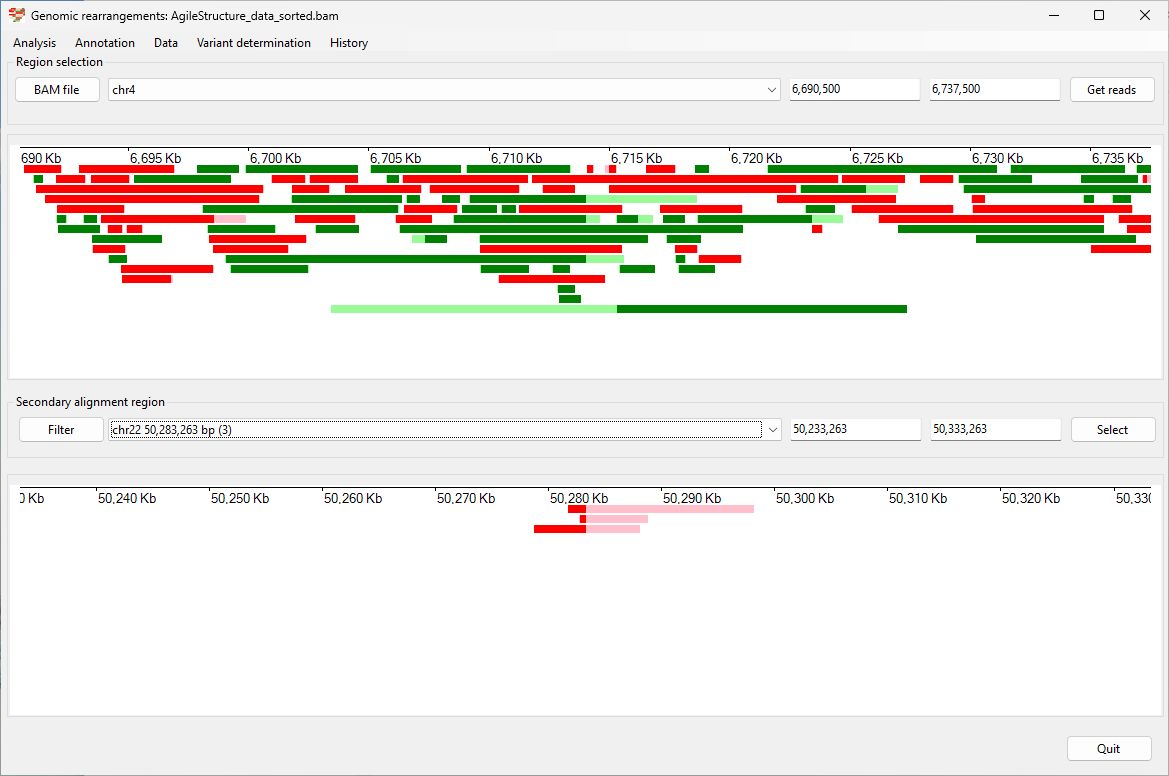


Figure 5a. The visualisation of all reads mapping to the breakpoint in a translocation


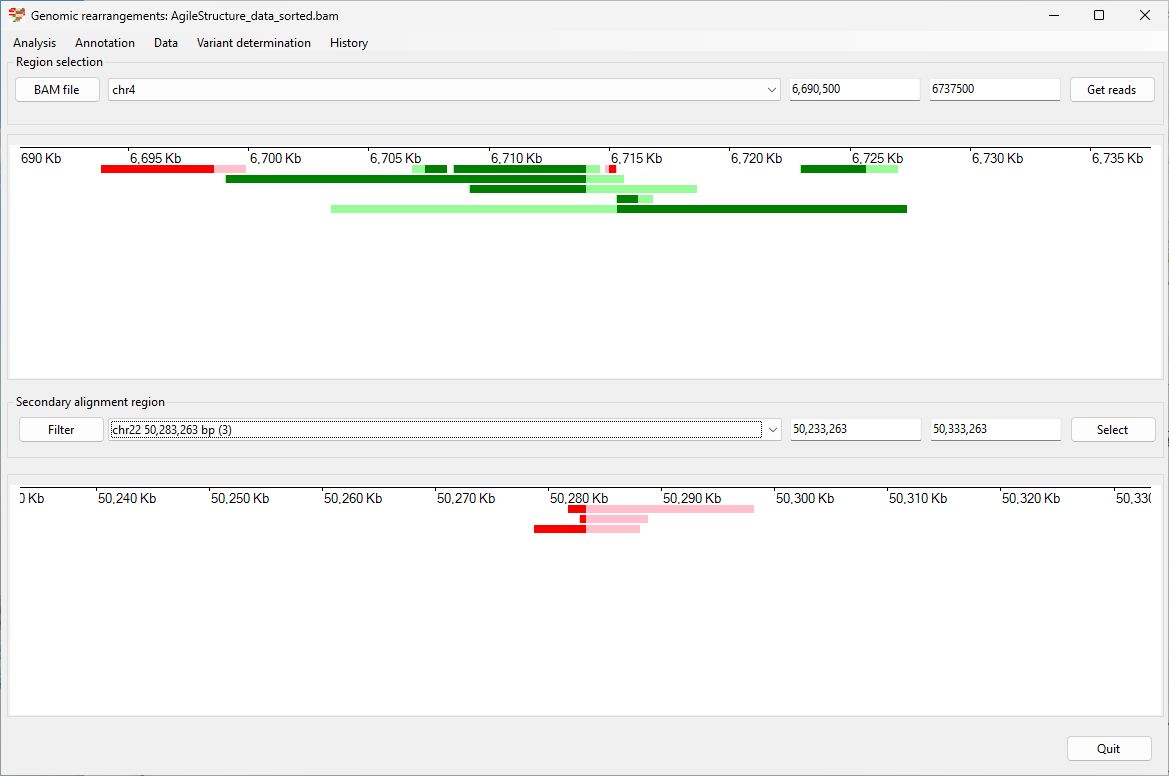


Figure 5b. The visualisation of split reads mapping to the breakpoint in a translocation


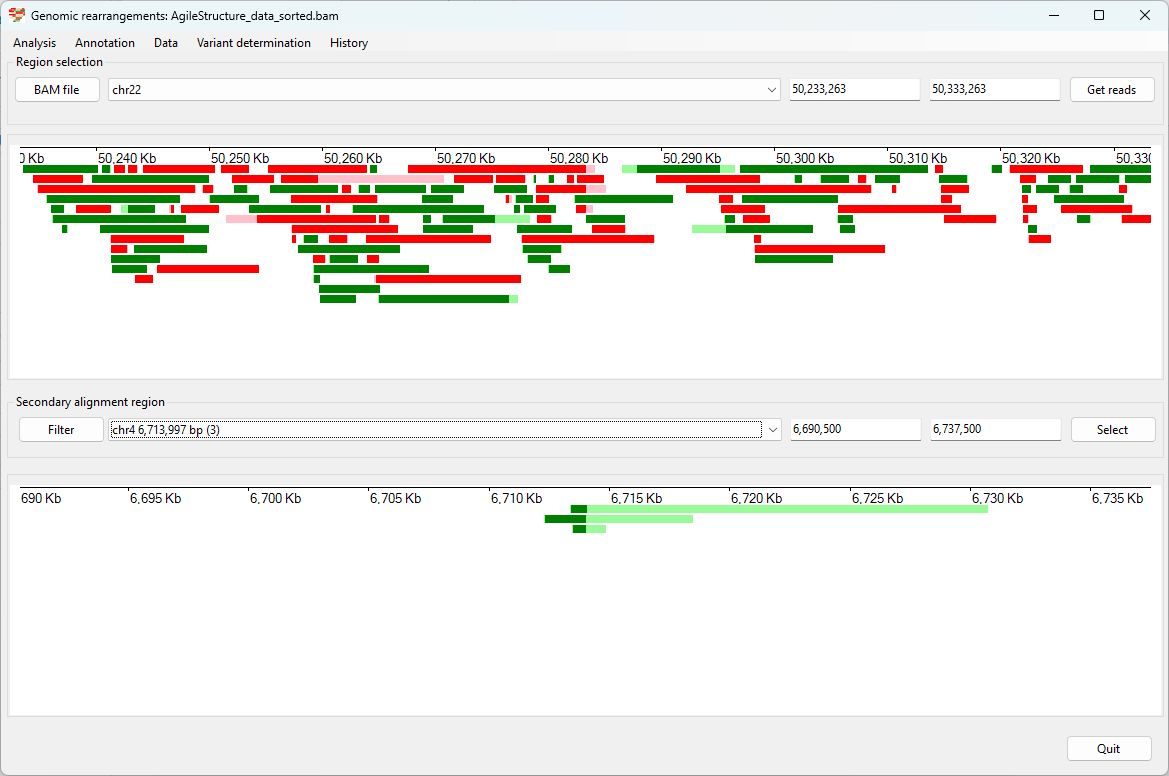


Figure 5c. The visualisation of all reads mapping to the reciprocal breakpoint in a translocation


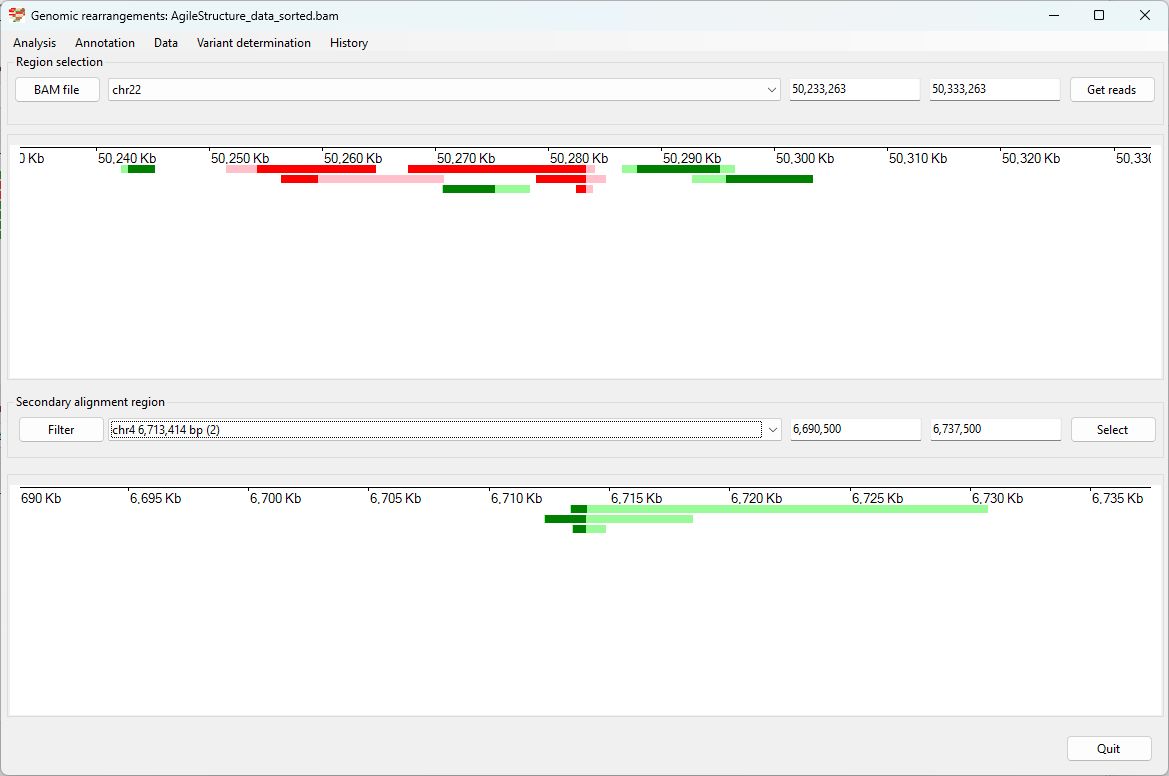


Figure 5d. The visualisation of split reads mapping to the reciprocal breakpoint in a translocation

### Ring Chromosomes

The arrangement of the primary and secondary alignments is identical to those seen in a tandem duplication. Consequently, it is not possible to distinguish between a ring chromosome and a tandem duplication without secondary information, such as a karyotype or copy number data.


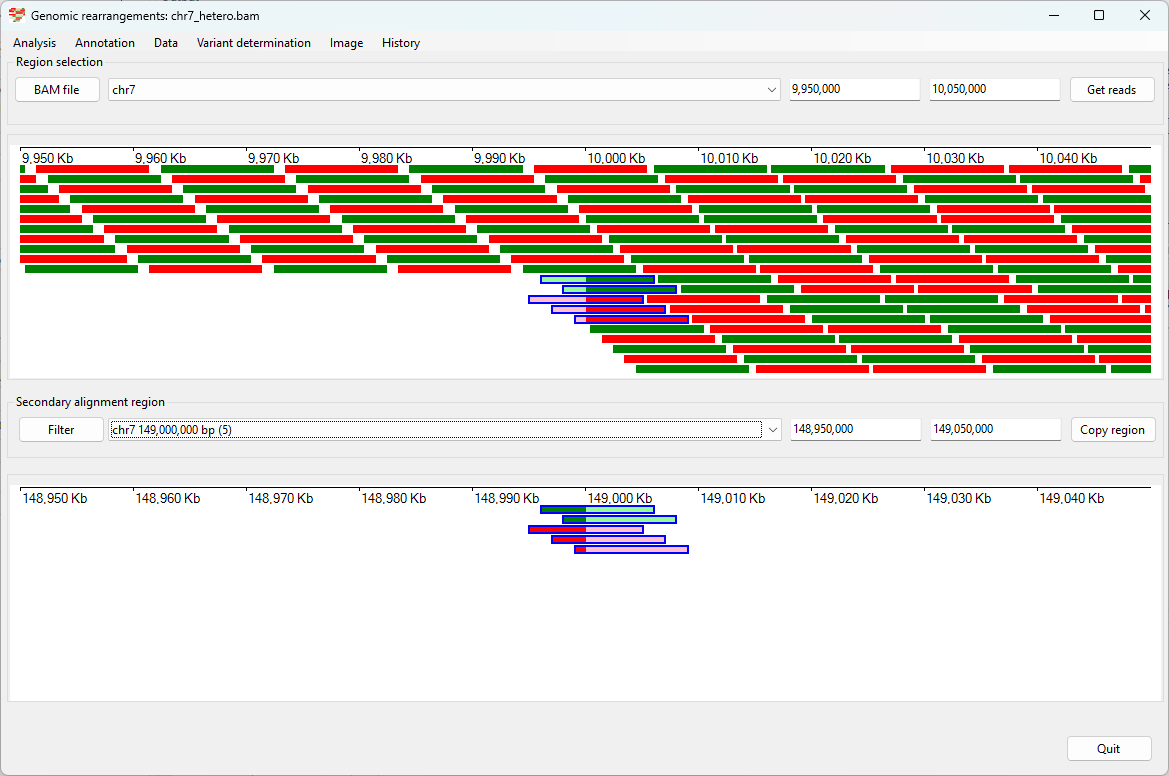


Figure 6a. The visualisation of all reads mapping to the 5’ breakpoint of a synthetic ring


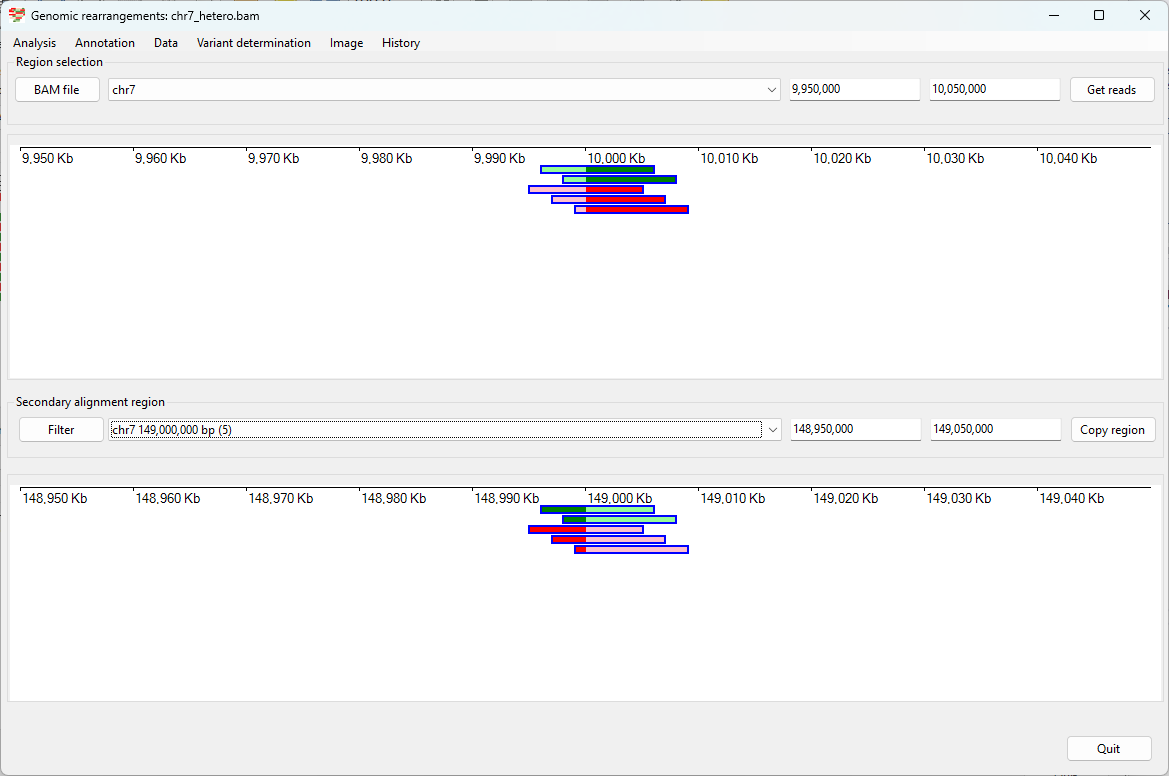


Figure 6b. The visualisation of split reads mapping to the 5’ breakpoint of a synthetic ring


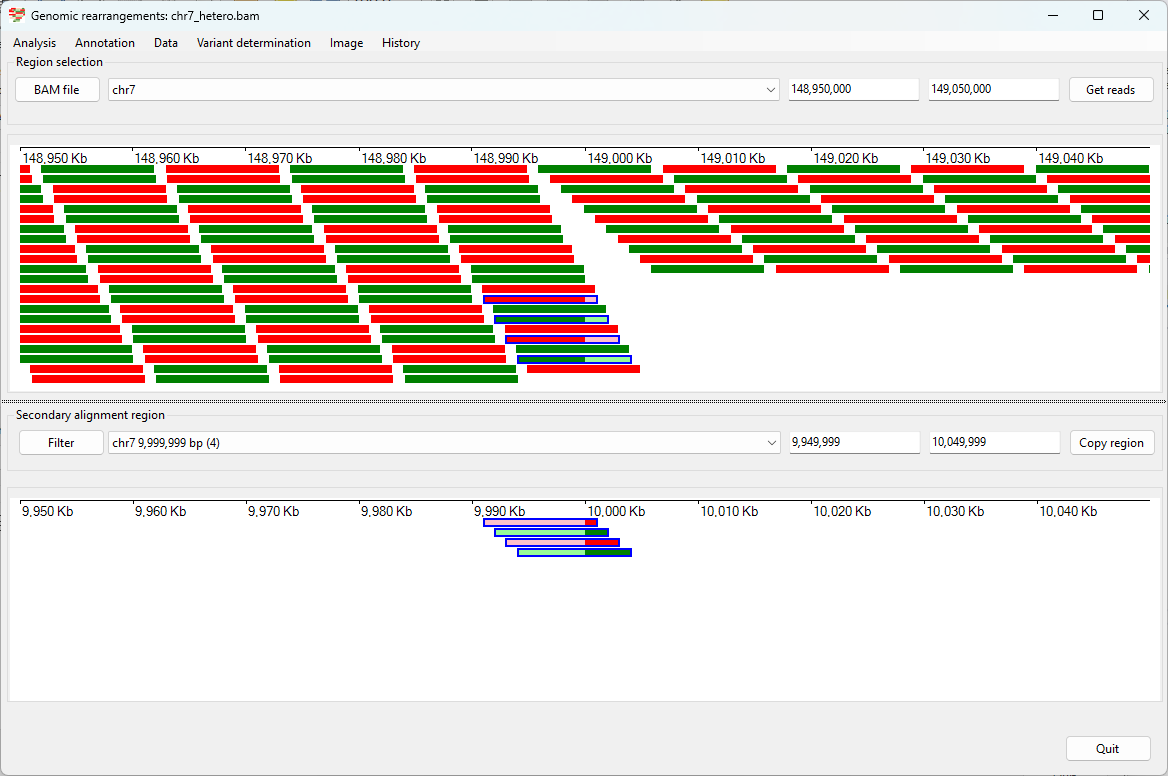


Figure 6c. The visualisation of all reads mapping to the 3’ breakpoint of a synthetic ring

**
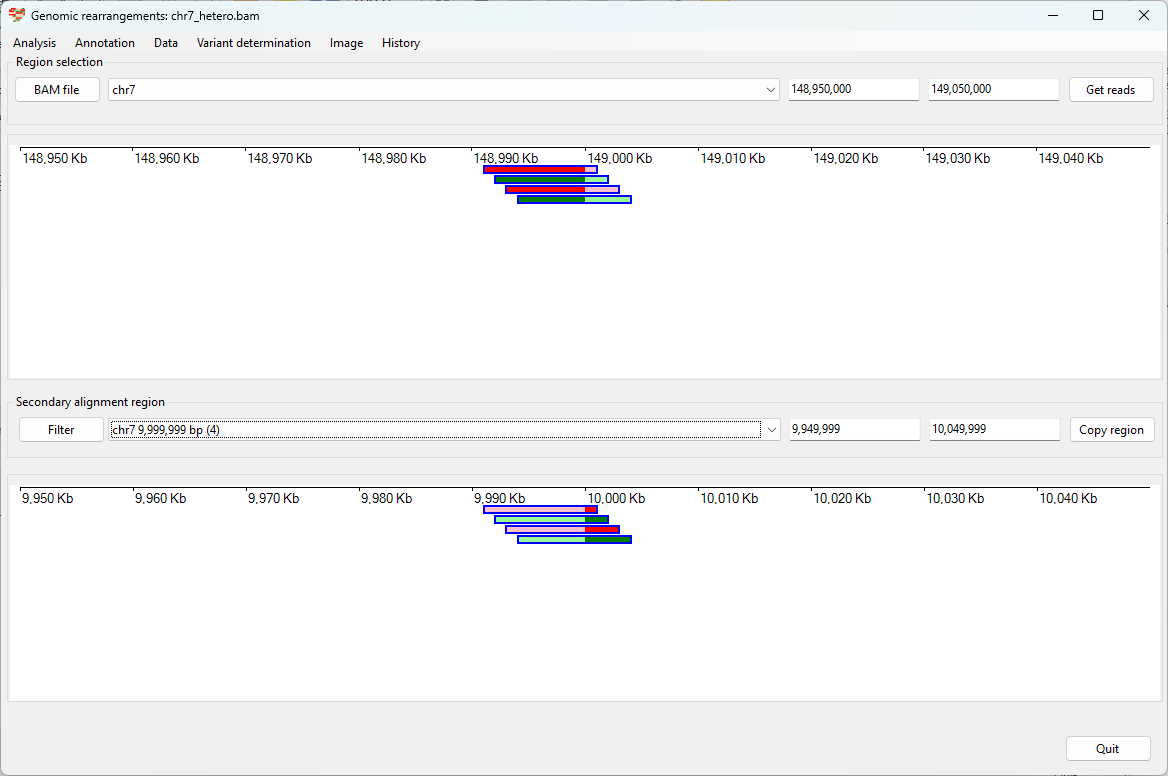
**

Figure 6d. The visualisation of split reads mapping to the 3’ breakpoint of a synthetic ring

## Insertion and deletion identification using gapped reads

### Deletions:

Deletions are shown as two blocks of alignment linked by a thing black line. The breakpoints (indicated by the black and blue arrow) are located at the point where the thin horizontal black line touches the rectangles representing the alignments.

### Insertions:

Insertions can be identified as a position containing a series of thin vertical lines transecting blocks of alignment (purple arrow)


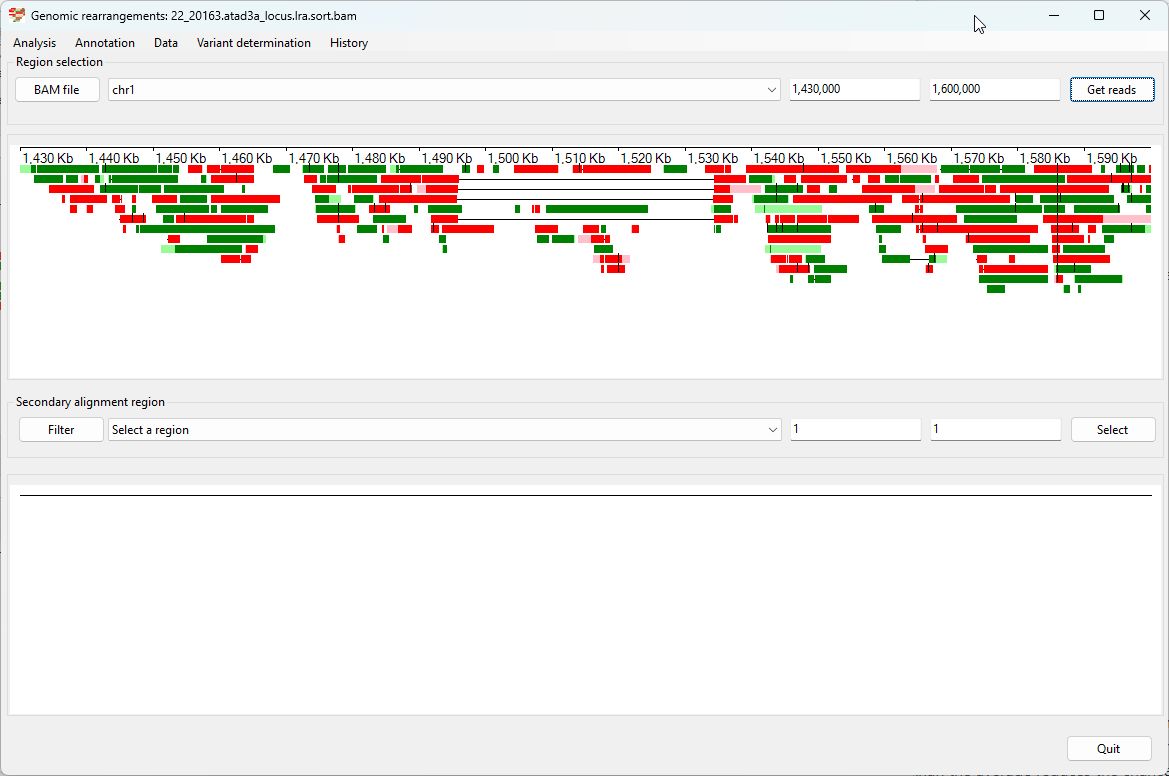


Figure 7a. The visualisation of all reads mapping to a region containing a deletion and an insertion


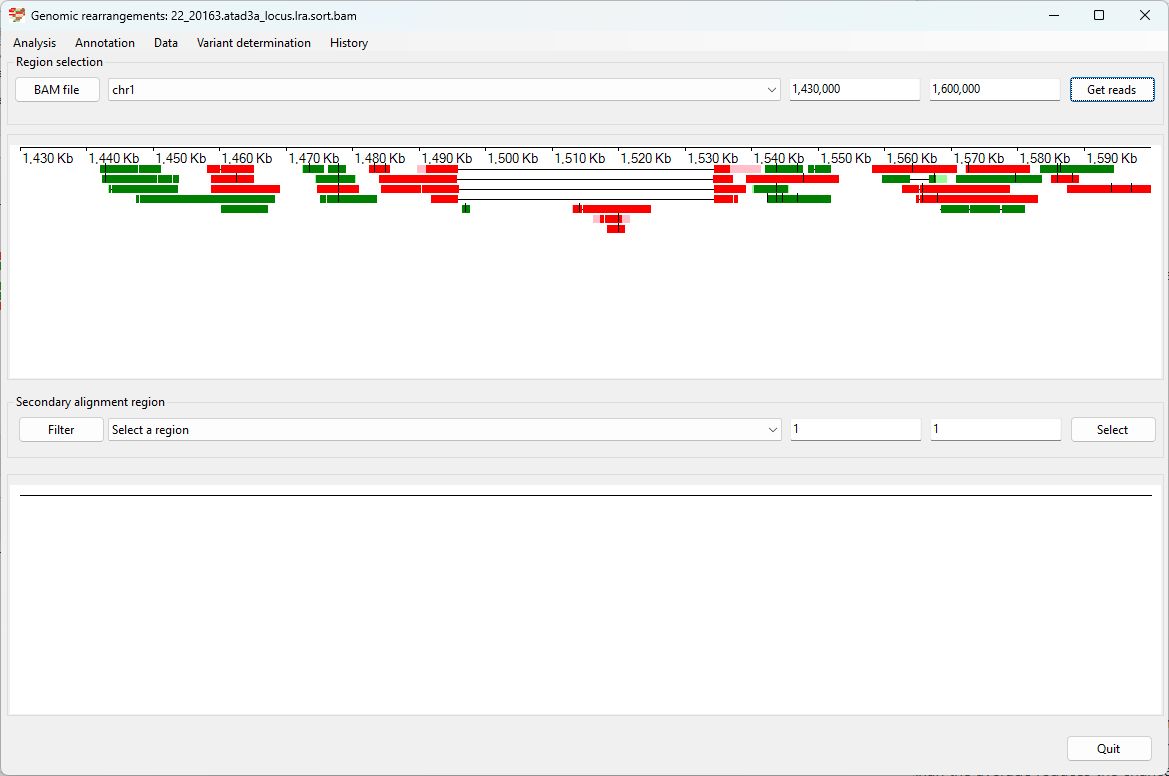


Figure 7b. The visualisation of reads containing gapped reads that suggest an insertion or deletion that map to a region containing a deletion and an insertion

## Cause of variation in number of split read columns


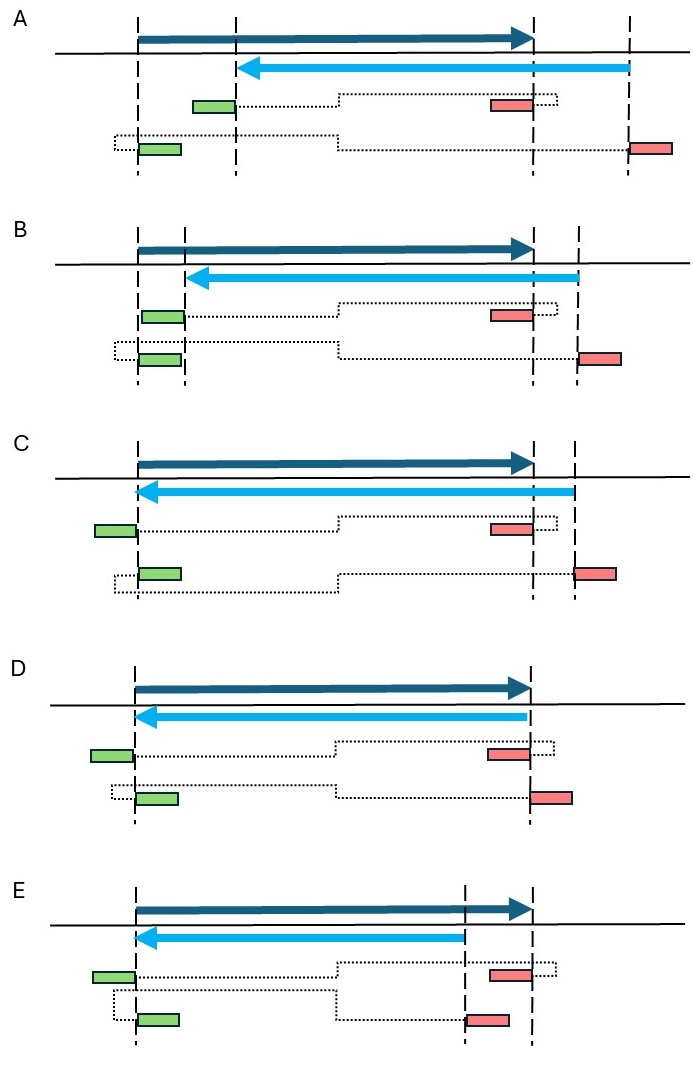


Figure 8: The dark blue arrow marks the extent of the rearranged sequence, while the pale blue arrow marks the extent of the sequence replaced by the copied sequence. The green and red rectangles indicate the location of a read’s alignments, with the different colours only indicating the orientation of the alignment differs. The dotted line indicates how a read’s alignments are arranged within the read. The vertical bashed line marks the apparent breakpoint at each column of split reads.

When analysing rearrangements, its apparent that they fall in three main groups, which have 2, 3 or 4 distinct columns of split reads. Rearrangements with 2 columns (or insertions with 3 columns) are classed as simple and can be annotated by AgileStructure, using one of its specific functions. Those with 3 or 4 columns should be annotated with the *Complex rearrangement* window.

Why a rearrangement as 2, 3 or 4 columns of split reads depends on whether sequence is lost or duplicated at the break points. Figures 8a and 8b show an offset inversion with the size of the offset in Figures 8a greater than Figures 8b. It can be seen that as the size of the duplicated/deleted sequences decreases the distances between the two columns of split reads decreases. In Figures 8c no sequence was deleted/duplicated at 5’ side of the arrangement, consequently, the first two columns of split reads have merged to form a single column. It should be noted that the pattern of the read’s alignments has not changed. In Figures 8d, no sequence is duplicated or deleted at either side of the rearrangement and so only two columns of split reads are present. Again, should be noted that the pattern of the read’s alignments has not changed. Finally. In Figure 8e the deleted sequence is shorter than the inserted sequences resulting in a duplication of sequences at the 3’ end of the inversion. This results in the end of the rearrangement been delimited by two columns of split reads, but unlike Figures 8a and 8b, the alignments lay between the columns. Yet again, should be noted that the pattern of each read’s alignments has not changed.

# Supplementary Tables

### Table S1

| Rearrangement | Number read breakpoints | Primary and secondary alignment orientation | Location of a read’s alignments relative to the read breakpoints |
| --- | --- | --- | --- |
| Deletion | 2 | +/+ or -/- | Both external |
| Inversion | 2 | +/- or -/+ | One is internal, and the other is external to the inverted sequence. |
| Ring chromosome | 2 | +/+ or -/- | Both alignments are found between the breakpoints. |
| Tandem duplication | 2 | +/+ or -/- | Both alignments are found between the breakpoints |
| Inverted Duplication  (5’ insertion site) | 2 | +/- or -/+ | Either both in the duplicated sequence or both out. The 5’ position will contain 3 times the number of alignments as the 3’ position. These extra reads will have overlapping primary and secondary alignments. |
| Inverted Duplication  (3’ insertion site) | 2 | +/- or -/+ | Split-reads with alignments at each side of the duplication have alignments that are either both within or both outside the breakpoints. The 3’ position will contain 3 times the number of alignments as the 5’ position. The extra alignments will have primary and secondary alignments on either side of the 3’ breakpoint. |
| Insertion | 3 | Either all +/+ or -/-  or all +/- or -/+ | One alignment is present at the insertion site, and the other maps to one end of the copied sequence’s original site. |
| Translocation | 2 | No set pattern, but the orientation will be consistent between reads with the same alignment pattern. | One alignment will map to one chromosome, while its linked alignment will map to the other chromosome. If the translocation is balanced, reads will map to either side of the breakpoints. However, if it is unbalanced, reads will only map to one side of the breakpoint. |

Table S1 Overview of alignment characteristics for each type of rearrangement. These descriptions refer to simple variants, see the text for a discussion of more complex variants.

### Table S2

| Inserted at: | Inserted sequence | Insert inverted | Analysis function | Ambiguous Annotation | Description |
| --- | --- | --- | --- | --- | --- |
| See legend | See legend | No | Ring chromosome | Yes | Ring chromosome |
| chr7:43,600,000-43,750,000 | None | - | Deletion | No | Deletion |
| chr7:43,600,000 | chr7:43,600,000-43,750,000 | No | Duplication | Yes | Tandem duplication |
| chr7:43,600,000 | chr7:43,600,000-43,750,000 | Yes | Duplication | No | Inverted Duplication (5’ insertion) |
| chr7:43,740,000-43,750,000 | chr7:43,600,000-43,750,000 | No | Duplication | No | Duplication in which 10 Kb of the 3' end of the original sequence is lost |
| chr7:43,750,000 | chr7:43,600,000-43,750,000 | No | Duplication | No | Tandem duplication |
| chr7:43,750,000 | chr7:43,600,000-43,750,000 | Yes | Duplication | No | Inverted Duplication (3’insertion) |
| chr7:20,000,000 | chr8:43,600,000-43,750,000 | No | Insertion | No | Insertion into different chromosome |
| chr7:20,000,000 | chr7:43,600,000-43,750,000 | Yes | Insertion | No | The reverse complement of a sequence is inserted on a different chromosome |
| chr7:20,000,000 | chr8:43,600,000-43,750,000 | Yes | Insertion | No | The reverse complement of a sequence is inserted on a different chromosome |
| chr7:43,600,000 | chr8:43,600,000-43,750,000 | No | Insertion | No | Insertion into different chromosome |
| chr7:43,600,000 | chr8:43,600,000-43,750,000 | Yes | Insertion | No | The reverse complement of a sequence is inserted on a different chromosome |
| chr7:50,000,000 | chr8:43,600,000-43,750,000 | No | Insertion | No | Insertion into different chromosome |
| chr7:50,000,000 | chr7:43,600,000-43,750,000 | Yes | Insertion | No | The reverse complement of a sequence is inserted on a different chromosome |
| chr7:50,000,000 | chr8:43,600,000-43,750,000 | Yes | Insertion | No | The reverse complement of a sequence is inserted on a different chromosome |
| chr8:20,000,000 | chr7:43,600,000-43,750,000 | No | Insertion | No | Insertion in to same chromosome |
| chr8:20,000,000 | chr7:43,600,000-43,750,000 | Yes | Insertion | No | The reverse complement of a sequence is inserted on a different chromosome |
| chr8:50,000,000 | chr7:43,600,000-43,750,000 | No | Insertion | No | Insertion in to same chromosome |
| chr8:50,000,000 | chr7:43,600,000-43,750,000 | Yes | Insertion | No | The reverse complement of a sequence is inserted on a different chromosome |
| chr7:43,600,000-43,750,000 | chr7:43,600,000-43,750,000 | Yes | Inversion | No | Inversion |
| chr7:1-3,000,000 | chr8:1-4,000,000 | No | Translocation | No | Unbalanced translocation in which the p telomere of chromosome 8 is replaced by the p telomere of chromosome 7 |
| chr7:1-3,000,000, chr8:1-4,000,000 | chr8:1-4,000,000, chr7:1-3,000,000 | No | Translocation | No | Balanced translocation in which the p telomeres are exchanged between chromosomes 7 and 8 |
| chr7:1-3,000,000 | chr8:141,138,635-145,138,635 | Yes | Translocation | No | Unbalanced translocation in which the p telomere of chromosome 7 is replaced by the q telomere of chromosome 8 |
| chr7:1-3,000,000, chr8:141,138,635-145,138,635 | chr8:141,138,635-145,138,635, chr7:1-3,000,000 | Yes | Translocation | No | Balanced translocation in which the p telomere of chromosome 7 is exchanged for the q telomere of chromosome 8 |
| chr7:156,345,972-159,345,97 | chr8:141,138,635-145,138,635 | No | Translocation | No | Unbalanced translocation in which the q telomere of chromosome 7 is replaced by the q telomere of chromosome 8 |
| chr7:156,345,972-159,345,97, chr8:141,138,635-145,138,635 | chr8:141,138,635-145,138,635, chr7:156,345,972-159,345,97 | No | Translocation | No | Balanced translocation in which the q telomeres are exchanged between chromosomes 7 and 8 |
| chr7:156,345,972-159,345,97 | chr8:1-4,000,000 | Yes | Translocation | No | Unbalanced translocation in which the q telomere of chromosome 7 is replaced by the p telomere of chromosome 8 |
| chr7:156,345,972-159,345,97, chr8:1-4,000,001 | chr8:1-4,000,000, chr7:156,345,972-159,345,97 | Yes | Translocation | No | Balanced translocation in which the q telomere of chromosome 7 is exchanged for the p telomere of chromosome 8 |
| chr8:1-4,000,000 | chr7:1-3,000,000 | No | Translocation | No | Unbalanced translocation in which the p telomere of chromosome 7 is replaced by the p telomere of chromosome 8 |
| chr8:1-4,000,000 | chr7:156,345,972-159,345,97 | Yes | Translocation | No | Unbalanced translocation in which the p telomere of chromosome 8 is replaced by the q telomere of chromosome 7 |
| chr8:141,138,635-145,138,635 | chr7:156,345,972-159,345,97 | No | Translocation | No | Unbalanced translocation in which the q telomere of chromosome 8 is replaced by the q telomere of chromosome 7 |
| chr8:141,138,635-145,138,635 | chr7:1-3,000,000 | Yes | Translocation | No | Unbalanced translocation in which the q telomere of chromosome 8 is replaced by the p telomere of chromosome 7 |
| chr7:20,000,000-21,000,000 | chr7:43,600,000-43,750,000 | No | Complex rearrangement | No | Deletion of sequence at the site of an insertion. The inserted sequence originated from a site significantly 5' to the insertions site |
| chr7:20,000,000-21,000,000 | chr8:43,600,000-43,750,000 | No | Complex rearrangement | No | A chromosome 8 sequence is inserted into chromosome 7 with sequence loss at insertion site |
| chr7:20,000,000-21,000,000 | chr7:43,600,000-43,750,000 | Yes | Complex rearrangement | No | The reverse complement of a sequence is inserted significantly 5' of the copied sequence |
| chr7:20,000,000-21,000,000 | chr8:43,600,000-43,750,000 | Yes | Complex rearrangement | No | The reverse complement of a chromosome 8 sequence is inserted into chromosome 7 with sequence loss at insertion site |
| chr7:43,550,000-43,700,000 | chr7:43,600,000-43,750,000 | Yes | Complex rearrangement | Yes | Inversion in which 50 kb of 5' flanking sequence lost while 50 kb of the original 3' sequence is retained |
| chr7:43,550,000-43,750,000 | chr7:43,600,000-43,750,000 | Yes | Complex rearrangement | No | Inversion in which 50 Kb of 5' flanking sequence is lost |
| chr7:43,550,000-43,800,000 | chr7:43,600,000-43,750,000 | Yes | Complex rearrangement | No | Inversion on which 50 kb of sequence each side of the inversion is lost |
| chr7:43,590,000 | chr7:43,600,000-43,750,000 | No | Complex rearrangement | No | Tandem duplication with 10 kb of unduplicated sequence between the duplicated sequences. |
| chr7:43,590,000-43,600,000 | chr7:43,600,000-43,750,000 | No | Complex rearrangement | No | Tandem duplication in which 10 kb of the original sequence is deleted at the insertion site which is at the 5' end of the original sequence |
| chr7:43,590,000-43,600,000 | chr7:43,600,000-43,750,000 | Yes | Complex rearrangement | No | Inverted duplication in which the insertion deletes 10 kb of the 5' end of the original sequence |
| chr7:43,600,000-43,610,000 | chr7:43,600,000-43,750,000 | No | Complex rearrangement | No | Tandem duplication in which the 10 Kb of the 5' end of the original sequence is deleted |
| chr7:43,600,000-43,750,000 | chr8:43,600,000-43,750,000 | No | Complex rearrangement | No | A chromosome 8 sequence is inserted into chromosome 7 with sequence loss at insertion site |
| chr7:43,600,000-43,610,000 | chr7:43,600,000-43,750,000 | Yes | Complex rearrangement | No | Inverted duplication in which the insertion deletes 10 kb of the 5' end of the original sequence |
| chr7:43,600,000-43,700,000 | chr7:43,600,000-43,750,000 | Yes | Complex rearrangement | No | Inversion in which 50 Kb of the 3' end of the original sequence is retained |
| chr7:43,600,000-43,800,000 | chr7:43,600,000-43,750,000 | Yes | Complex rearrangement | No | Inversion in which 50 kb of the 3' flanking sequence is lost |
| chr7:43,600,000-43,750,000 | chr8:43,600,000-43,750,000 | Yes | Complex rearrangement | No | The reverse complement of a chromosome 8 sequence is inserted into chromosome 7 with sequence loss at insertion site |
| chr7:43,610,000 | chr7:43,600,000-43,750,000 | No | Complex rearrangement | No | Duplication with 10 kb of unduplicated sequence between the duplicated sequences. |
| chr7:43,610,000 | chr7:43,600,000-43,750,000 | No | Complex rearrangement | No | Tandem duplication in which the insertion within the 3' end of original sequence |
| chr7:43,610,000 | chr7:43,600,000-43,750,000 | Yes | Complex rearrangement | No | Inverted duplication in which insertion is within the copied sequence |
| chr7:43,650,000-43,700,000 | chr7:43,600,000-43,750,000 | Yes | Complex rearrangement | Yes | Inversion in which 50 Kb of sequence at the 5' and 3' ends of the copied sequence is retained |
| chr7:43,650,000-43,750,000 | chr7:43,600,000-43,750,000 | yes | Complex rearrangement | Yes | Inversion in which 50 Kb of the copied sequence retained at the 5' side of the inversion |
| chr7:43,650,000-43,800,000 | chr7:43,600,000-43,750,000 | Yes | Complex rearrangement | Yes | Inversion in which 50 Kb of the 5' end of the copied sequence is retained and 50 kb of 3' flanking sequence is deleted |
| chr7:43,740,000 | chr7:43,600,000-43,750,000 | No | Complex rearrangement | No | Tandem duplication in which the insertion within the 5' end of original sequence |
| chr7:43,740,000 | chr7:43,600,000-43,750,000 | Yes | Complex rearrangement | No | Inverted duplication in which the inserted sequences is located within the 3' end of the copied sequence |
| chr7:43,750,000-43,760,000 | chr7:43,600,000-43,750,000 | No | Complex rearrangement | No | Tandem duplication in which 10 kb of the original sequence is deleted at the insertion site which is at the 3' end of the original sequence |
| chr7:43,760,000 | chr7:43,600,000-43,750,000 | Yes | Complex rearrangement | Yes | Inverted duplication in which the inserted sequences is 10 Kb 3' to the copied sequences |
| chr7:50,000,000-51,000,000 | chr7:43,600,000-43,750,000 | No | Complex rearrangement | No | Deletion of sequence at the site of an insertion. The inserted sequence originated from a site significantly 3' to the insertions site |
| chr7:50,000,000-51,000,000 | chr8:43,600,000-43,750,000 | No | Complex rearrangement | No | A chromosome 8 sequence is inserted into chromosome 7 with sequence loss at insertion site |
| chr7:50,000,000-51,000,000 | chr7:43,600,000-43,750,000 | Yes | Complex rearrangement | No | The reverse complement of a sequence is inserted significantly 3' of the copied sequence |
| chr7:50,000,000-51,000,000 | chr8:43,600,000-43,750,000 | Yes | Complex rearrangement | No | The reverse complement of a chromosome 8 sequence is inserted into chromosome 7 with sequence loss at insertion site |
| chr8:20,000,000-21,000,000 | chr7:43,600,000-43,750,000 | No | Complex rearrangement | No | A chromosome 7 sequence is inserted into chromosome 8 with sequence loss at insertion site |
| chr8:20,000,000-21,000,000 | chr7:43,600,000-43,750,000 | Yes | Complex rearrangement | No | The reverse complement of a sequence is inserted on a different chromosome with sequence loss at insertion site |
| chr8:43,600,000-43,610,000 | chr7:43,600,000-43,750,000 | No | Complex rearrangement | No | A chromosome 7 sequence is inserted into chromosome 8 with sequence loss at insertion site |
| chr8:43,600,000-43,750,000 | chr7:43,600,000-43,750,000 | Yes | Complex rearrangement | No | The reverse complement of a sequence is inserted on a different chromosome with sequence loss at insertion site |
| chr8:50,000,000-51,000,000 | chr7:43,600,000-43,750,000 | No | Complex rearrangement | No | A chromosome 7 sequence is inserted into chromosome 8 with sequence loss at insertion site |
| chr8:50,000,000-51,000,000 | chr7:43,600,000-43,750,000 | Yes | Complex rearrangement | No | The reverse complement of a sequence is inserted on a different chromosome with sequence loss at insertion site |

Table S2 legend: If a single position is noted in the ‘Inserted at:’ column no sequences was deleted at the insertion site. ‘Analysis function’ indicates which analysis menu option was selected (Variant determination > Use soft clipped data > option). If the value in the ‘Ambiguous Annotation’ column is ‘Yes’ the rearrangement could not be resolved without secondary data. The synthetic ring chromosome was created by appending the chr7: 10,000,000 to 60,000,000 sequences to the end of the chr7: 60,000,001 to 149,000,000 sequences. Synthetic reads originating from this junction would map to the standard reference sequence as if they originated form a ring chromosome breakpoint.

### Table S3

| Rearrangement | Reference | Origin | Variant |
| --- | --- | --- | --- |
| Deletion | Watson et al. 2014 | Publication | chr7:146,534,699_146,611,541del |
|  |  | This work | chr7.146,534,703_146,914,542del |
| Duplication | Sailer et al. 2021 | Publication | chr12:37,206,133–37,300,425dup |
|  |  | This work | chr12.37,206,133_37,300,424dup |
| Inversion | Watson et al. 2016 | Publication | chr7.27,762,423_93,599,530inv |
|  |  | This work using primary alignments at chr7, 27,762,427 | chr7.27,762,424_93,599,530inv |
|  |  | This work using primary alignments at chr7, 93,599,530 | chr7.27,762,424_93,599,530inv |
| Translocation | Hu et al. 2020 | Publication | t(chr6;chr8) (g.167,281,717:g.113,696,089) |
|  |  | This work using primary alignments on chromosome 6 | t(chr6;chr8) (g.167,281,716;g.113,696,100) |
|  |  | This work using primary alignments at chromosome 8 | t(chr6;chr8) (g.167,281,719;g.113,696,098) |

Table S3: List of variant annotations reported in the original work compared to those identified by AgileStructure

#### Table S3 references

Watson CM, Crinnion LA, Tzika A, Mills A, Coates A, Pendlebury M, Hewitt S, Harrison SM, Daly C, Roberts P, Carr IM, Sheridan EG, Bonthron DT. Diagnostic whole genome sequencing and split-read mapping for nucleotide resolution breakpoint identification in CNTNAP2 deficiency syndrome. Am J Med Genet A. 2014;164A:2649-55.

Sailer S, Coassin S, Lackner K, Fischer C, McNeill E, Streiter G, Kremser C, Maglione M, Green CM, Moralli D, Moschen AR, Keller MA, Golderer G, Werner-Felmayer G, Tegeder I, Channon KM, Davies B, Werner ER, Watschinger K. When the genome bluffs: a tandem duplication event during generation of a novel Agmo knockout mouse model fools routine genotyping. Cell Biosci. 2021;11:54.

Watson CM, Crinnion LA, Harrison SM, Lascelles C, Antanaviciute A, Carr IM, Bonthron DT, Sheridan E. A Chromosome 7 Pericentric Inversion Defined at Single-Nucleotide Resolution Using Diagnostic Whole Genome Sequencing in a Patient with Hand-Foot-Genital Syndrome. PLoS One. 2016;11:e0157075.

Hu L, Liang F, Cheng D, Zhang Z, Yu G, Zha J, Wang Y, Xia Q, Yuan D, Tan Y, Wang D, Liang Y, Lin G. Location of Balanced Chromosome-Translocation Breakpoints by Long-Read Sequencing on the Oxford Nanopore Platform. Front Genet. 2020;10:1313.

### Table S4

| Program | Language (and dependencies) | Type | Calls variants | Data source | Aligners | Graphical output | Purpose | Target | Published | Last updated |
| --- | --- | --- | --- | --- | --- | --- | --- | --- | --- | --- |
| Sniffles2  (Smolka 2024) | Python | Command line | Yes | ONT and PacBio | Minimap2 | No | Variant caller | Whole genome | 2024 | Recent |
| SVJedi-graph  (Romain 2023) | Python (minigraph) | Command line | Yes | ONT and PacBio | Minimap3 | No | Variant caller | Whole genome | 2023 | 3 years |
| cuteSV  (Jiang 2020) | Ptyphon (scipy, pysam, Biopython, cigar, numpy, pyvcf3, scikit-learn) | Command line | Yes | ONT and PacBio | Minimap2, NGMLR, PBMM2 | No | Variant caller | Whole genome | 2020 | Recent |
| IGV  (Robinson 2011, Robinson 2017) | Java | Desktop | No | ONT and PacBio | NA | Yes | Data display | Targeted regions | 2017 | Recent |
| JBrowse2  (Diesh 2023) | TypeScript/JavaScript (MobX-state-tree, WebAssembly (WASM), Web Workers, Node.js. Desktop also needs react and Electron) | Web server, R package, command line or Desktop | No | NA | NA | Yes | Visualise aligned data and compare genome assemblies | Targeted regions and whole genomes | 2023 | Continuous |
| nanoSV  (Cretu 2017) | Python | Command line | Yes | ONT | BWA, Minimap2, NGMLR | No | Variant caller | Whole genome | 2017 | 7 years |
| PBHoney  (English 2014) | Python | Command line | Yes | PacBio | not stated | No | Variant caller | Whole genome | 2014 | 6 years |
| Ribbon  (Nattestad 2021) | JavaScript | Web server | Yes | Not stated | not stated | Yes | Variant caller and read visualisation | Whole genome | 2021 | 2 years |
| SVHunter  (Gao 2025) | Python (numpy, pandas, TensorFlow, pysam, math, scikit-learn) | Command line | Yes | ONT and PacBio | not stated | No | Variant caller | Whole genome | 2025 | 1 year |
| SVTopo  (Belyeu 2025) | Rust/Python/JavaScript | Command line | No | PacBio | pbmm2 | Yes | Visualise aligned read data | Targeted regions | 2025 | Recent |
| SVhawkeye  (Xiao 2024) | Python, R, Perl | Command line with linked graphical viewer | No | NA | NA | Yes | Visualise aligned read data | Targeted regions | 2024 | 2 years |
| AgileStructure | C# .net (.net 6 or later) | Desktop | Yes | ONT and PacBio | Full functionality: Minimap2. Reduced functionality: lra | Yes | Targeted variant caller and data display | Targeted regions | 2026 | Recent |

Table S4: Comparison of programs used for visualisation and/or variant calling of aligned long‑read sequencing data. Information on software dependencies, data sources, and preferred aligners is taken from each tool’s GitHub repository or published manuscript and may not be exhaustive.

#### Table S4 references

Smolka M., Paulin L.F., Grochowski C.M. et al. (2024) Detection of mosaic and population-level structural variants with Sniffles2. Nat. Biotechnol., 42, 1571–1580.
Romain S., Lemaitre C. (2023) SVJedi-graph: improving the genotyping of close and overlapping structural variants with long reads using a variation graph. Bioinformatics, 39 (Suppl. 1), i270–i278.
Jiang T., Liu Y., Jiang Y. et al. (2020) Long-read-based human genomic structural variation detection with cuteSV. Genome Biol., 21, 189.
Robinson J.T., Thorvaldsdóttir H., Winckler W. et al. (2011) Integrative genomics viewer. Nat. Biotechnol., 29, 24–26.
Robinson J.T., Thorvaldsdóttir H., Wenger A.M., Zehir A., Mesirov J.P. (2017) Variant review with the Integrative Genomics Viewer. Cancer Res., 77, e31–e34.
Diesh, C., Stevens, G.J., et al. (2023)  JBrowse 2: a modular genome browser with views of synteny and structural variation. Genome Biol., 24, 74.
Cretu Stancu M., van Roosmalen M.J., Renkens I. et al. (2017) Mapping and phasing of structural variation in patient genomes using nanopore sequencing. Nat. Commun., 8, 1326.
English A.C., Salerno W.J., Reid J.G. (2014) PBHoney: identifying genomic variants via long-read discordance and interrupted mapping. BMC Bioinformatics, 15, 180.
Nattestad M., Aboukhalil R., Chin C.-S., Schatz M.C. (2021) Ribbon: intuitive visualization for complex genomic variation. Bioinformatics, 37, 413–415.
Gao R., Hu H., Jiang Z. et al. (2025) SVHunter: long-re- ad-based structural variation detection through the transformer model. Brief. Bioinform., 26, bbaf203.
Belyeu J.R., Rowell W.J., Lake J.A. et al. (2025) Complex structural variant visualization with SVTopo. BMC Genomics, 26, 903.
12 Xiao Y., Yu T., Liang F., Hou T. (2024) SVHawkeye: an ultra-fast software for user-friendly visualization of targeted structural fragments from BAM files. Front. Genet., 15, 1352443.
